# Overproduction of mycotoxin biosynthetic enzymes triggers *Fusarium* toxisome-shaped structure formation via endoplasmic reticulum remodeling

**DOI:** 10.1371/journal.ppat.1011913

**Published:** 2024-01-02

**Authors:** Minhui Wang, Ningjie Wu, Huiyuan Wang, Chang Liu, Qiaowan Chen, Tianming Xu, Yun Chen, Youfu Zhao, Zhonghua Ma

**Affiliations:** 1 State Key Laboratory of Rice Biology, Key Laboratory of Biology of Crop Pathogens and Insects, Institute of Biotechnology, Zhejiang University, Hangzhou, People’s Republic of China; 2 Zhejiang Research Institute of Chemical Industry, Hangzhou, People’s Republic of China; 3 Irrigated Agriculture Research and Extension Center, Department of Plant Pathology, Washington State University, Prosser, Washington, United States of America; Purdue University, UNITED STATES

## Abstract

Mycotoxin deoxynivalenol (DON) produced by the *Fusarium graminearum* complex is highly toxic to animal and human health. During DON synthesis, the endoplasmic reticulum (ER) of *F*. *graminearum* is intensively reorganized, from thin reticular structure to thickened spherical and crescent structure, which was referred to as “DON toxisome”. However, the underlying mechanism of how the ER is reorganized into toxisome remains unknown. In this study, we discovered that overproduction of ER-localized DON biosynthetic enzyme Tri4 or Tri1, or intrinsic ER-resident membrane proteins FgHmr1 and FgCnx was sufficient to induce toxisome-shaped structure (TSS) formation under non-toxin-inducing conditions. Moreover, heterologous overexpression of Tri1 and Tri4 proteins in non-DON-producing fungi *F*. *oxysporum* f. sp. *lycopersici* and *F*. *fujikuroi* also led to TSS formation. In addition, we found that the high osmolarity glycerol (HOG), but not the unfolded protein response (UPR) signaling pathway was involved in the assembly of ER into TSS. By using toxisome as a biomarker, we screened and identified a novel chemical which exhibited high inhibitory activity against toxisome formation and DON biosynthesis, and inhibited *Fusarium* growth species-specifically. Taken together, this study demonstrated that the essence of ER remodeling into toxisome structure is a response to the overproduction of ER-localized DON biosynthetic enzymes, providing a novel pathway for management of mycotoxin contamination.

## Author summary

The mycotoxin deoxynivalenol (DON) produced by the *Fusarium graminearum* complex is the most frequently detected mycotoxin in cereal grains worldwide. DON is synthesized in the compartmentalized organelle named “DON toxisome” that is highly remodeled, organized smooth endoplasmic reticulum (OSER). In this study, we demonstrated that ER remodeling into toxisome structure in *F*. *graminearum* is a response to overproduction of ER-localized DON biosynthetic enzymes, including Tri1 and Tri4. We further found that the HOG signaling pathway is important for the toxisome-shaped structure assembly. Moreover, using the Tri1-GFP labeled toxisome as a biomarker, we identified a novel small chemical exhibiting high inhibitory activity on toxisome assembly and DON biosynthesis. Our study elucidates the intrinsic mechanism of ER remodeling into toxisomes upon DON induction, and blocking the ER remodeling would provide a new avenue for management of FHB and mycotoxin contamination.

## Introduction

*Fusarium graminearum* is an aggressive fungal pathogen causing Fusarium head blight (FHB), which is an economically devastating disease of cereal crops especially wheat [1,2]. Due to global warming and changes in cultural practices, FHB has frequently reached epidemic levels in wheat-growing regions worldwide during the past 20 years, resulting in enormous yield losses across millions of hectares [3,4,5]. In addition to severe yield and economic losses, *F*. *graminearum* produces various mycotoxins during infection of wheat, such as trichothecenes (including deoxynivalenol (DON) and its acetylated derivatives) and zearalenone, thus raising food safety risks and posing a great threat to human and animal health [4]. Among these mycotoxins, DON is the most frequently detected mycotoxin in cereal grains all over the world, with an average incidence rate more than 50% [4]. DON inhibits eukaryotic protein synthesis by binding to the ribosome and may cause emesis, diarrhea, anorexia and immunedysregulation [6,7]. Consequently, many countries have set maximum permissible levels for DON in cereals and cereal products to protect consumers from mycotoxicosis [8,9]. In addition, DON is a key virulence factor that promotes *F*. *graminearum* infection on wheat plants [10]. Therefore, understanding the biosynthesis and regulation of DON in *F*. *graminearum* is critical for combatting FHB and mycotoxin contamination.

Trichothecenes are a large family of toxic sesquiterpenoid secondary metabolites (SMs) produced by certain species of *Fusarium* and other fungal genera [11]. Trichothecenes produced by *F*. *graminearum* include DON, nivalenol (NIV), and acetylated derivatives 15-ADON and 3-ADON [4]. The trichothecene biosynthetic gene (*TRI*) cluster is one of the most studied SM gene clusters in fungi. In *F*. *graminearum*, the biosynthesis of trichothecene involves 15 *TRI* genes, which are located on three different chromosomes in *F*. *graminearum*: a 12-gene core *TRI* cluster on chromosome 2, two genes at the *TRI1*-*TRI16* locus on chromosome 1, and the single-gene *TRI101* locus on chromosome 3 [4,12,13]. Trichothecene biosynthesis begins with the cyclization of the primary metabolite farnesyl pyrophosphate, which is catalyzed by the trichodiene synthase Tri5, resulting in the product trichodiene (TDN). TDN is subsequently oxidized by cytochrome P450 monooxygenase Tri4 to yield isotrichotriol. Further reactions sequentially catalyzed by Tri101, Tri11 and Tri3 convert isotrichotriol to calonectrin, which is hydroxylated by cytochrome P450 monooxygenase Tri1 to generate 7,8-dihydroxycalonectrin (7,8-DHC). The following transformations convert 7,8-DHC to 3-ADON or 15-ADON, which is deacetylated by Tri8, leading to the formation of DON [4,12,14]. In addition to genes encoding trichothecene biosynthetic enzymes, the *F*. *graminearum TRI* cluster also encodes a predicted major facilitator superfamily (MFS) transporter Tri12, and two cluster-specific transcription regulators Tri6 and Tri10 [15,16,17]. Because trichothecene is a secondary metabolite, the *TRI* cluster genes are not expressed in toxin non-inducing conditions, while their expressions are highly induced during incubation in toxin-inducing medium or during infection on plants [4].

Enzymes for fungal secondary metabolite (SM) synthesis are often compartmentalized at conserved subcellular sites, which plays important roles in precursor channeling, concentration of biosynthetic components, sequestering and trafficking pathway intermediates and products, and promoting pathway efficiency [14,18]. In *Aspergillus*, aflatoxin biosynthetic enzymes Nor-1, Ver-1 and Vbs initially reside in cytoplasm and then relocate to motile vesicles termed aflatoxisomes for aflatoxin biosynthesis [19,20,21]. In *Penicillium chrysogenum*, the biosynthesis site of penicillin shifts from cytoplasm to the peroxisome for the formation of final product [22]. In *A*. *fumigatus* and *A*. *nidulans*, melanin biosynthetic enzymes involved in the early steps are recruited to endosomes to facilitate melanogenesis, while late melanin enzymes accumulate in the cell wall [23]. Recent studies have suggested that subcellular compartmentalization also occurs during DON biosynthesis in *F*. *graminearum* [24,25]. Menke and colleagues first observed that under DON-inducing conditions, two cytochrome P-450 oxygenases (Tri4 and Tri1) responsible for catalyzing early and late steps in trichothecene biosynthesis in *F*. *graminearum* were co-localized to ∼3 μm spherical organelles called “toxisomes”, which were presumed to be the site of trichothecene assembly [26]. Later, it was discovered that toxisomes are highly remodeled, organized smooth endoplasmic reticulum (OSER) with pronounced expansion at perinuclear- and peripheral positions [24]. Interestingly, under non-DON-inducing conditions, the native perinuclear and peripheral ER in *F*. *graminearum* appeared reticulate and thin as determined by ER marker proteins GFP-HDEL, Hmr1-GFP and Sec22-GFP, while the ER was remodeled to highly thickened spherical, crescent and ovoid toxisomes upon DON induction, indicating a striking remodeling of the ER structure under toxin inducing conditions [24]. The remodeling of ER (*e*.*g*. toxisome formation) appears to be critical for trichothecene production since inhibition of toxisome formation by the fungicide phenamacril, which targets the motor protein myosin I, leads to significant reduction in DON accumulation [25]. However, the molecular mechanism of how the ER is reorganized into toxisomes remains elusive.

The objective of this study was to explore the underlying mechanism of toxisome formation under DON-inducing conditions. Our results showed that overproduction of ER-localized SM enzymes Tri4 or Tri1, or intrinsic ER-resident membrane proteins FgHmr1 and FgCnx was sufficient to induce toxisome-shaped structure (TSS) formation, even under non-toxin-inducing conditions. Interestingly, heterologous overexpression of Tri1 and Tri4 proteins in trichothecene non-production fungi *F*. *oxysporum* f. sp. *lycopersici* and *F*. *fujikuroi* also leads to TSS formation. In addition, the UPR signaling pathway appears to be unnecessary for the remodeling of ER into TSS, whereas the HOG signaling pathway is important for the TSS assembly. Importantly, using the Tri1-GFP labeled toxisome as a biomarker, we identified a novel compound ZJU212 with high inhibitory activity on toxisome assembly and DON biosynthesis, providing new ways for management of DON contamination and FHB.

## Results

### Overexpressed Tri proteins induce toxisome-shaped structure formation under toxin non-inducing conditions

**Fig 1:**
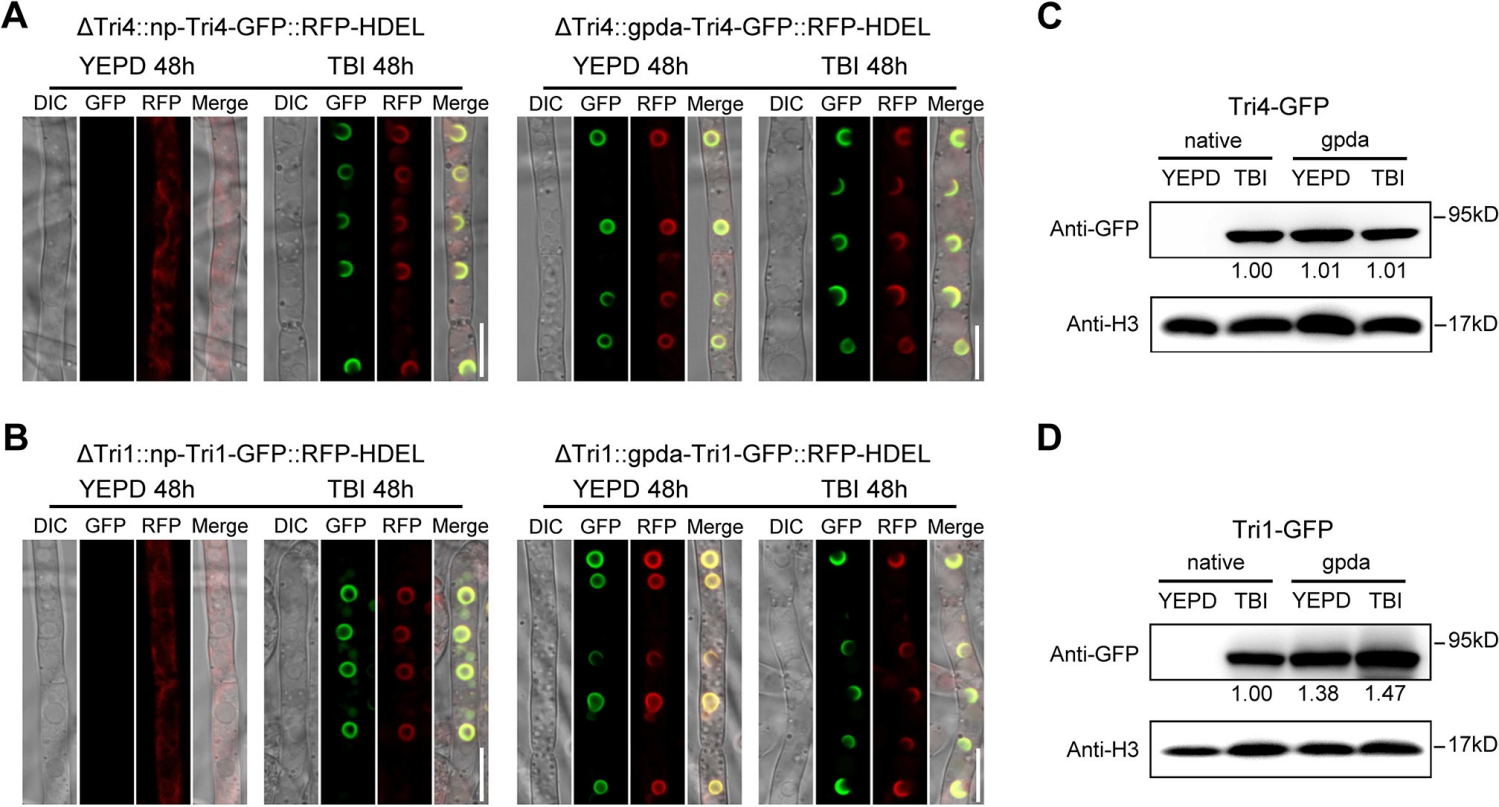
Overproduction of Tri4 and Tri1 induced toxisome-shaped structure (TSS) formation under DON non-inducing conditions. (**A-B**) Localization of Tri4-GFP (A) and Tri1-GFP (B) under native promoter (left panels) and gpda promoter (right panels) in YEPD medium or TBI medium. RFP-HDEL is used as the ER marker. Images of each strain were taken after incubation at 28°C for 48 h. DIC indicates differential interference contrast. Bar = 10 μm. (**C-D**) The protein abundance of Tri4-GFP (C) and Tri1-GFP (D) isolated from the same set of samples used in A and B was determined by western blot assay with the anti-GFP antibody. The protein abundance of H3 of each sample served as a loading control. The intensities of the western blot bands were quantified using the Image J software, and numbers below the bands represent relative intensity of GFP normalized to H3. Native and gpda represent Tri proteins that are expressed under native and gpda promoters, respectively.

In *F*. *graminearum*, the expression of trichodiene (Tri4) and calonectrin oxygenase (Tri1) enzymes that catalyze early and late steps of the trichothecene biosynthetic pathway is highly upregulated in trichothecene biosynthesis inducing (TBI) medium [4]. Both enzymes co-localized to DON toxisomes when *F*. *graminearum* was cultured in TBI medium for 48 h, when accumulation of Tri1 and Tri4 proteins reached peak levels [25]. However, at 20–24 h of incubation in TBI medium, expression of Tri1 and Tri4 protein was low, and Tri1-GFP or Tri4-GFP was observed as faint reticulate GFP pattern of the ER and no toxisome structures were observed [24]. Based on these observations, we hypothesized that remodeling of the ER into DON toxisome structure correlates with the overexpression of Tri1 and Tri4 proteins. To test this, Tri4-GFP and Tri1-GFP constructs with a strong constitutive promoter (the gpda promoter from *Aspergillus nidulans*) were generated and transformed into ΔTri4 and ΔTri1 mutants, respectively. The corresponding GFP fusion constructs with the native promoter (np) were used as controls. To visualize the ER structure, the ER marker RFP-HDEL expression vector was also constructed and transformed into the ΔTri4 and ΔTri1 strains. As shown in Fig 1A and 1B (left panels), Tri4- and Tri1-GFP driven by native promoters displayed no fluorescence signals in the DON non-inducing medium YEPD (yeast extract peptone dextrose) after 48 hours of incubation, while the ER labeled by RFP-HDEL showed a thin reticular structure. Whereas Tri4- and Tri1-GFP were highly induced and localized at the thickened spherical and crescent structures (toxisomes) in the TBI medium after incubation for 48 hours, and the toxisomes co-localized with the RFP-HDEL labelled ER (Fig 1A and 1B left panels). When Tri4- and Tri1-GFP were driven under the gpda promoter, the two proteins displayed toxisome localizations even in the YEPD medium after 48 h growth and localized to the remodeled ER (Fig 1A and 1B right panel). In the TBI medium, Tri4- and Tri1-GFP under the gpda promoter were similarly localized to toxisomes (Fig 1A and 1B right panel). Western blotting assay further showed that expression of the Tri4- or Tri1-GFP proteins driven by the native promoter strains in TBI medium was similar to those by the gpda promoter strains in YEPD medium (Fig 1C and 1D). Due to the ability of strains expressed under gpda promoter to form toxisome structure in YEPD, we next examined the DON production of these strains under DON non-inducing (YEPD) and inducing (TBI) conditions by LC-MS. Results showed that although overexpression of Tri4- and Tri1-GFP under gpda promoter induced the ER remodeling into spherical and crescent structures in YEPD medium (Fig 1A and 1B), the two strain ΔTri4::gpda-Tri4-GFP::RFP-HDEL and ΔTri1::gpda-Tri1-GFP::RFP-HDEL did not produce any DON toxin in YEPD medium, while produced DON normally in TBI (S1 Fig). Therefore, these Tri protein overexpression-induced spherical and crescent structures formed in non-inducing medium were not a functional “toxisome” with the ability for DON production, and thus were designated as “toxisome-shaped structure” (TSS). In addition, we also assessed the formation of TSS in another two commonly used nutrient-rich liquid media for *F*. *graminearum* growth, namely complete medium (CM) and potato dextrose broth (PDB). TSS was also observed in the strain ΔTri4::gpda-Tri4-GFP and ΔTri1::gpda-Tri1-GFP (under gpda promoter) after 48 h of incubation in CM and PDB media (S2 Fig), suggesting that formation of TSS is not related to the medium, but resulted from overexpression of Tri proteins. These results indicate that overproduction of Tri4 or Tri1 protein is sufficient for induction of ER remodeling to form TSS under DON non-inducing conditions.

### The transmembrane domain of Tri4 is essential for Tri4-marked TSS formation

**Fig 2:**
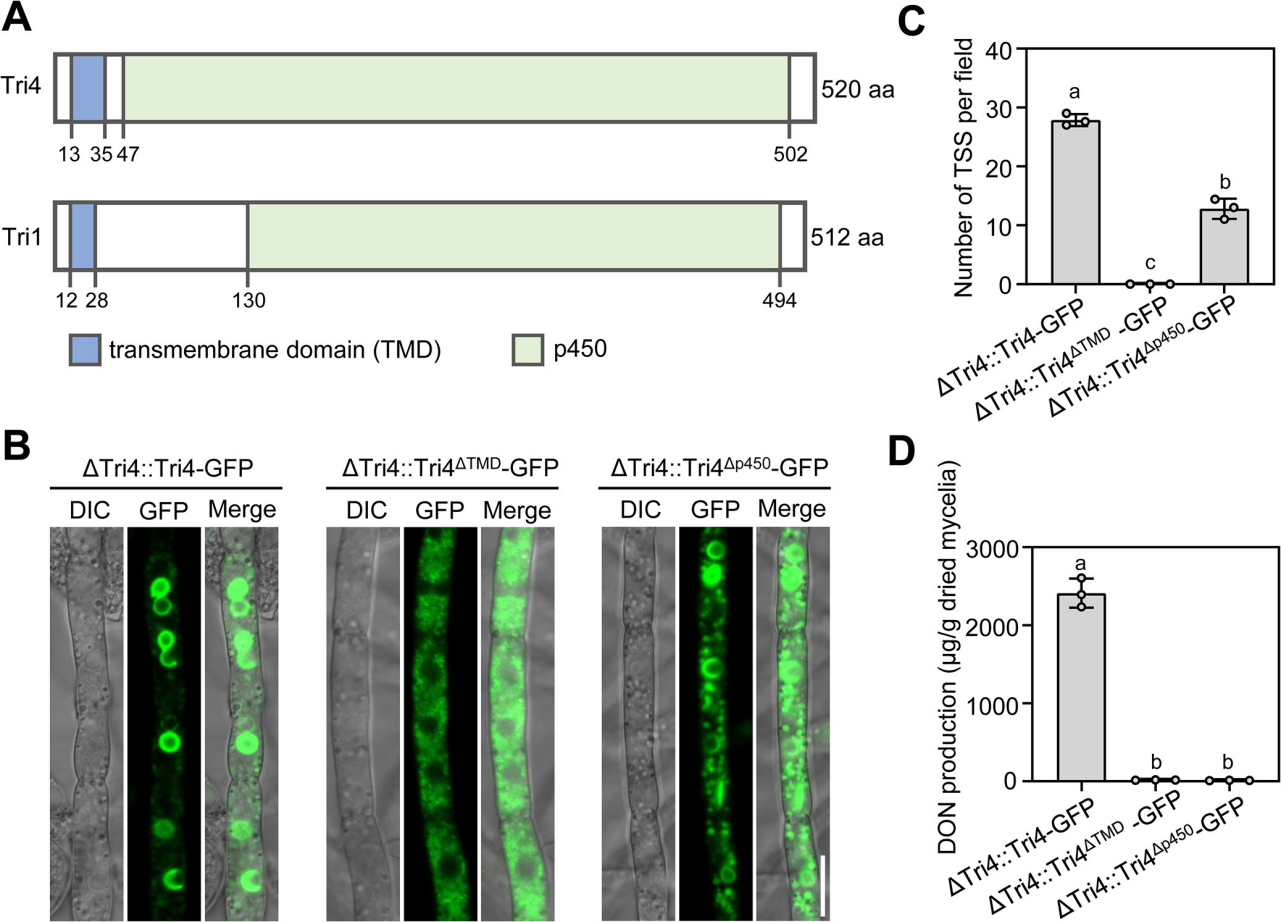
An essential role of the transmembrane domain of Tri4 in the formation of Tri4-marked TSS. (**A**) Domain architecture of Tri4 and Tri1. The number at the bottom indicates the deduced amino acid positions of corresponding domains. (**B**) The TSS formation patterns in truncated Tri4 proteins. Each strain was grown in TBI medium for 48 h. Bar = 10 μm. (**C**) The average number of toxisome-shaped structures in an examination field of 135 μm × 135 μm. (**D**) DON production of the truncated Tri4 complemented strains. After growth in TBI for 7 d, each strain was determined for DON production. Data represent the mean ± s.d. from three independent experiments. Different letters indicate a significant difference (*P <* 0.05) based on one-way ANOVA followed by Tukey’s multiple comparison test.

Tri4 and Tri1 are cytochrome P450 monooxygenases, each with a p450 catalytic domain at their C-terminus (Fig 2A). As predicted by InterPro [27], both Tri4 and Tri1 contain a putative short transmembrane domain (TMD) at their N-terminus (Fig 2A). To determine the role of TMD and p450 catalytic domains in toxisome-shaped structure formation, two truncated Tri4 constructs: the Tri4^ΔTMD^-GFP fusion construct (a truncated Tri4 lacking 13–35 aa fused with GFP) and Tri4^Δp450^-GFP fusion construct (a truncated Tri4 lacking 47–520 aa fused with GFP) under native promoter were generated and transformed into ΔTri4, respectively. The complemented strain ΔTri4::Tri4-GFP with full-length Tri4 was used as positive control. After 48 hours of incubation in TBI medium, the full-length Tri4-GFP was localized to the toxisomes, while the truncated Tri4^ΔTMD^-GFP protein was detected as diffuse fluorescent signals in the cytoplasm without any toxisome localization (Fig 2B). Interestingly, in the dual-labelled strain ΔTri4::Tri4^ΔTMD^-GFP::Tri1-RFP under native promoter, although the transmembrane-deleted Tri4^ΔTMD^-GFP protein was still localized dispersedly in the cytoplasm outside of toxisome, Tri1-RFP was localized to the toxisome-shaped structures (TSS) after 48 h incubation in TBI (S3 Fig), indicating that the transmembrane domain of Tri4 only affects the TSS labelled by Tri4, but not other protein like Tri1-induced TSS. The Tri4^Δp450^-GFP was partially localized to TSS, with some GFP signals simultaneously observed in cytoplasm (Fig 2B). However, the number of Tri4-marked TSS formed in the ΔTri4::Tri4^Δp450^-GFP strain decreased significantly as compared with the ΔTri4::Tri4-GFP strain (Fig 2B and 2C). These results indicate that the transmembrane domain of Tri4 is essential for Tri4-marked TSS formation while the p450 catalytic domain is partially required for Tri4-marked TSS formation. Consistent with TSS formation labelled by Tri4, the ΔTri4::Tri4^ΔTMD^-GFP strain was nearly unable to produce DON (Fig 2D), indicating that localization of Tri4 to the toxisome is critical for DON production.

### Overexpression of ER-resident proteins FgHmr1 and FgCnx is able to induce ER remodeling under toxin non-inducing conditions

**Fig 3:**
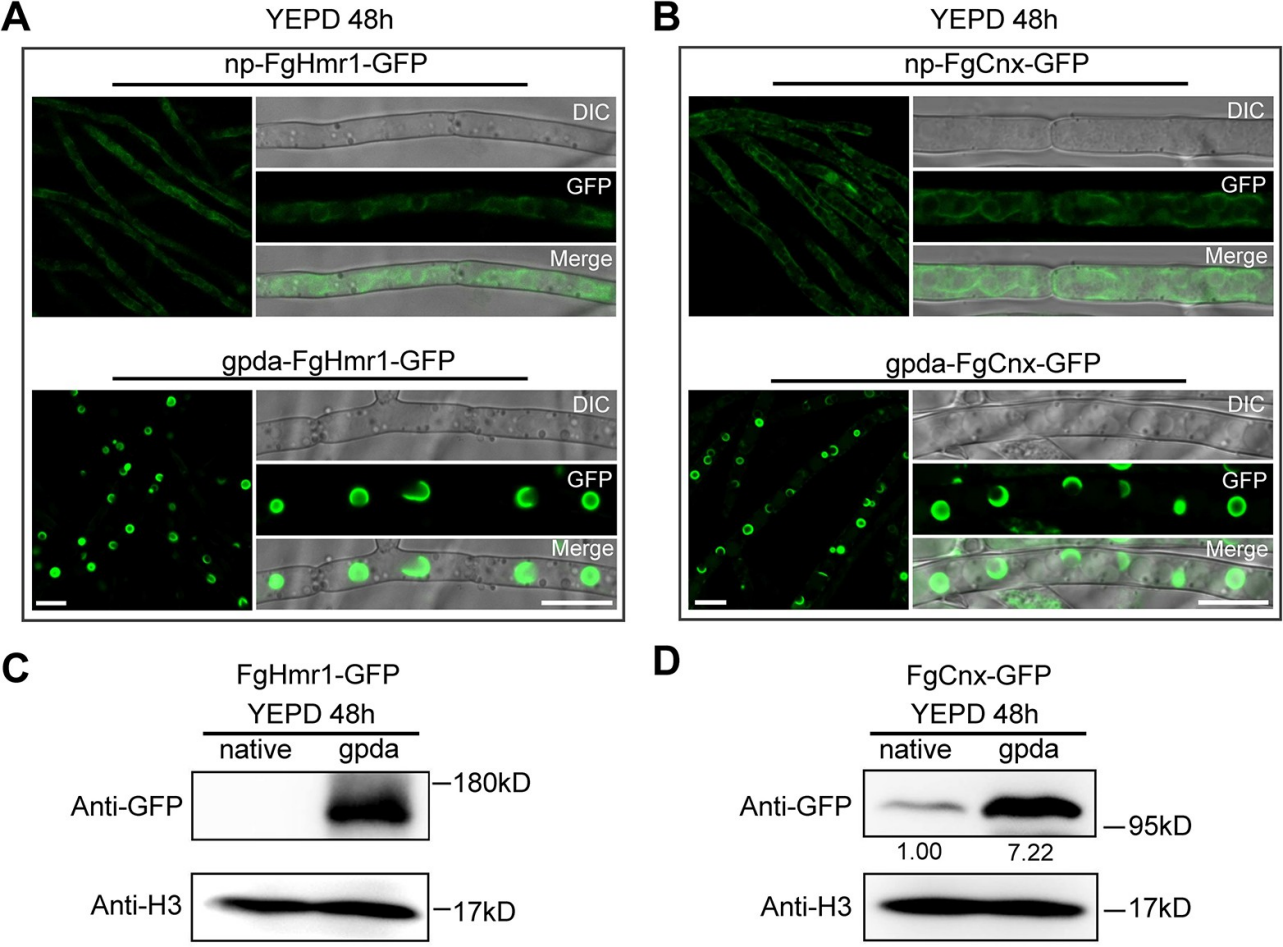
Overexpression of ER-resident proteins FgHmr1 and FgCnx triggers the ER remodeling into TSS. (**A-B**) Localization of FgHmr1-GFP (A) and FgCnx-GFP (B) under native promoter (upper panels) and gpda promoter (lower panels) in YEPD. Each strain was grown in YEPD medium at 28°C for 48 h. Bar = 10 μm. (**C-D**) The protein abundance of FgHmr1-GFP (C) and FgCnx-GFP (D) under native and gpda promoters was determined by western blot assay using the anti-GFP antibody. The protein H3 was used as a reference. The intensities of the western blot bands were quantified using the Image J software, and numbers below the bands represent relative intensity of GFP normalized to H3.

It has been reported that a few dozen ER resident enzymes can induce ER hypertrophy and reorganization when expressed at elevated levels [24]. Given that overexpression of ER-localized oxygenases Tri4 and Tri1 induced ER remodeling (Fig 1), we speculated whether overexpression of other ER-resident proteins would have similar effect. To test this, two ER-resident proteins FgHmr1 and FgCnx were selected for further investigation. Hmr1 (HMG-CoA reductase, a key enzyme in the isoprenoid biosynthetic pathway) and Cnx (calnexin, molecular chaperone of the ER) are two highly conserved integral membrane protein of ER in eukaryotes [28,29], and have been frequently used as ER markers in fungi and animals [30,31,32]. Different from Tri4 and Tri1, which are expressed only under specific induction conditions (such as TBI medium), Hmr1 and Cnx are constitutive expressed ER proteins. To determine effect of overproduction of these two proteins on ER remodeling, FgHmr1 and FgCnx were tagged with GFP at their C-terminal *in situ* to generate the strains np-FgHmr1-GFP and np-FgCnx-GFP driven by their native promoter (np), in which the np was further replaced with gpda strong promoter, yielding the overexpression strains gpda-FgHmr1-GFP and gpda-FgCnx-GFP. After incubation for 48 h in YEPD medium, fluorescent signal of FgHmr1-GFP or FgCnx-GFP under np was faint and observed at thin spherical (presumably perinuclear) and reticulate peripheral structures (Fig 3A and 3B, upper panel); while GFP fluorescent signals in the gpda-FgHmr1-GFP and gpda-FgCnx-GFP strains were significantly higher with thickened spherical and crescent structures (Fig 3A and 3B, lower panel), indicating a dramatic remodeling in ER structure. Western blotting assay further confirmed that the amount of FgCnx-GFP protein increased about 7 folds under the strong gpda promoter as compared to that under the np (Fig 3D). Consistently, the FgHmr1-GFP protein also increased significantly under the gpda promoter (Fig 3C). Interestingly, overexpression of another ER-localized membrane protein 14-α-demethylase FgCyp51A [33] in *F*. *graminearum* by the gpda promoter failed to induce ER remodeling into TSS (S4 Fig). Together, these results revealed that remodeling of ER into TSS can also be directly triggered by overproduction of certain ER-resident proteins in *F*. *graminearum*.

### ER remodeling in other fungi that do not produce DON

**Fig 4:**
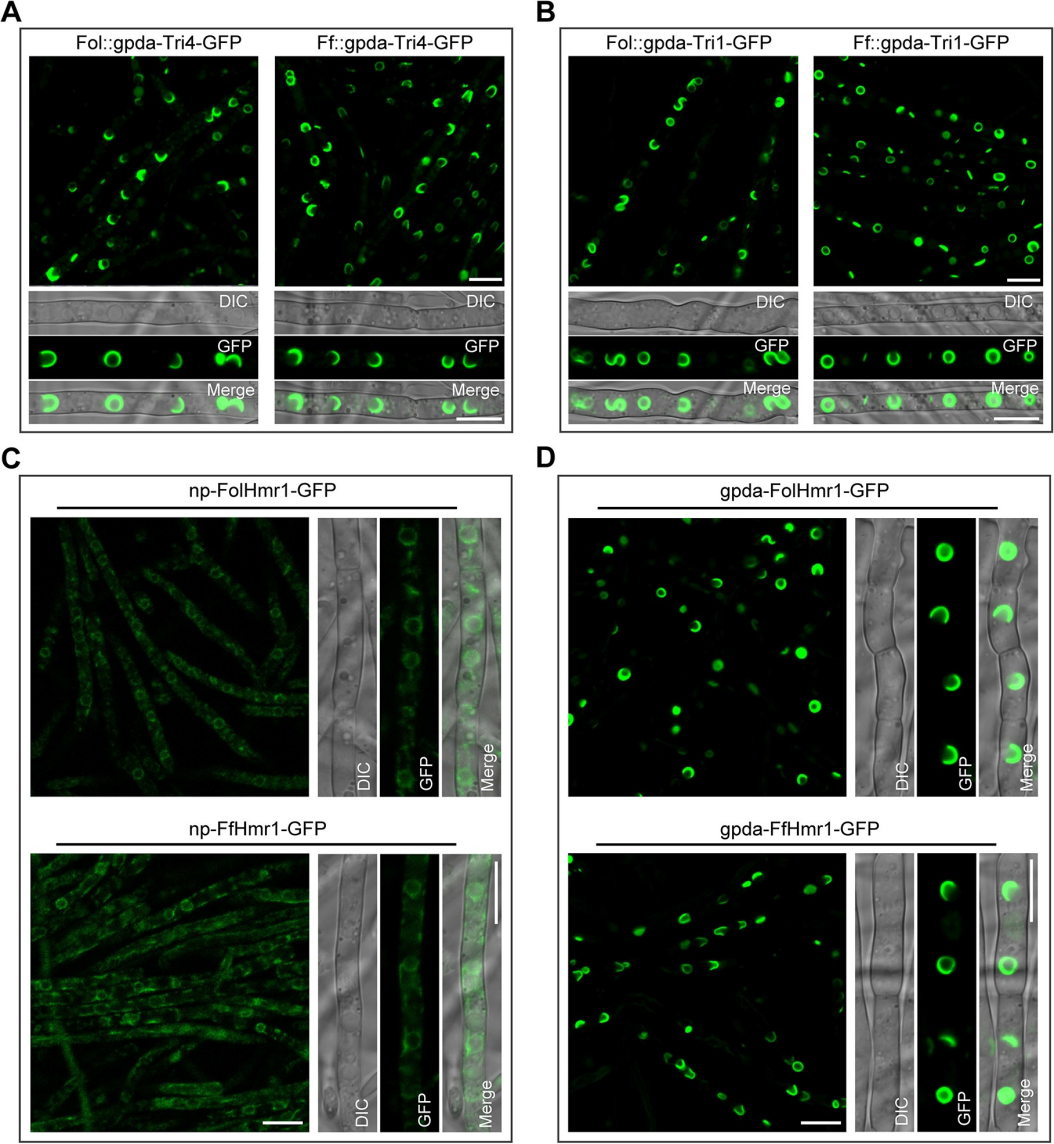
Remodeling of the ER in other fungi that do not produce DON. (**A-B**) Heterologous overexpression of Tri4-GFP (A) and Tri1-GFP (B) in *F*. *oxysporum* f. sp. *lycopersici* (*Fol*) (left panels) and *F*. *fujikuroi* (*Ff*) (right panels) induced the formation of TSS. Each strain was incubated in YEPD at 28°C for 48 h. Bar = 10 μm. (**C**) Localization of ER-resident protein Hmr1 under its native promoter in *Fol* (upper panel) and *Ff* (lower panel). Each strain was incubated in YEPD medium at 28°C for 48 h. Bar = 10 μm. (**D**) Upon overexpression of the endogenous Hmr1 in *Fol* (upper panel) and *Ff* (lower panel) by gpda promoter, the ER was reorganized into TSS. Images of each strain were taken after incubation in YEPD medium at 28°C for 48 h. Bar = 10 μm.

To further verify that formation of TSS in *F*. *graminearum* is triggered by overproduction of the DON biosynthetic enzymes, we preformed heterologous expression of Tri1 and Tri4 proteins in other filamentous fungi that do not produce DON. The Tri4-GFP or Tri1-GFP fusion construct under the strong gpda promoter identical to Fig 1 was transformed into *Fusarium oxysporum* f. sp. *lycopersici* (*Fol*) (causal agents of wilt disease of tomato) and *Fusarium fujikuroi* (*Ff*) (causal agents of bakanae of rice), respectively. It should be noted that these two phytopathogenic fungi don’t contain the DON biosynthetic gene cluster in their genomes. Results showed that both Tri4-GFP and Tri1-GFP were observed to be localized on the TSS in *Fol* and *Ff* (Fig 4A and 4B), indicating that high-level heterologous expression of the DON biosynthesis enzymes Tri1 or Tri4 in *Fol* and *Ff* is sufficient for induction of the ER remodeling to form TSS.

This observation also promoted us to ask whether homologous overproduction of ER-resident proteins in these two fungi would result in ER remodeling as well. To test this, endogenous ER-localized proteins FolHmr1 in *Fol* and FfHmr1 in *Ff* were fused with GFP at their C-terminals *in situ* to generate the strains np-FolHmr1-GFP and np-FfHmr1-GFP under their native promoters (np). Concurrently, the overexpression strains gpda-FolHmr1-GFP and gpda-FfHmr1-GFP, in which FolHmr1 and FfHmr1 were driven by the gpda promoter were also generated. When Hmr1-GFP was expressed under the np, both np-FolHmr1-GFP and np-FfHmr1-GFP displayed faint signals and localized at a thin perinuclear and reticulate peripheral ER (Fig 4C). Upon overexpression of Hmr1-GFP driven by the gpda promoter, thicker spheres and crescent ER structures were observed in both gpda-FolHmr1-GFP and gpda-FfHmr1-GFP strains (Fig 4D). These results demonstrated that homologous overproduction of the endogenous ER-resident protein Hmr1 in *Fol* and *Ff* can induce ER remodeling to form TSS.

### Regulation of the Tri4-marked TSS formation by the positive regulators in *TRI* cluster

**Fig 5:**
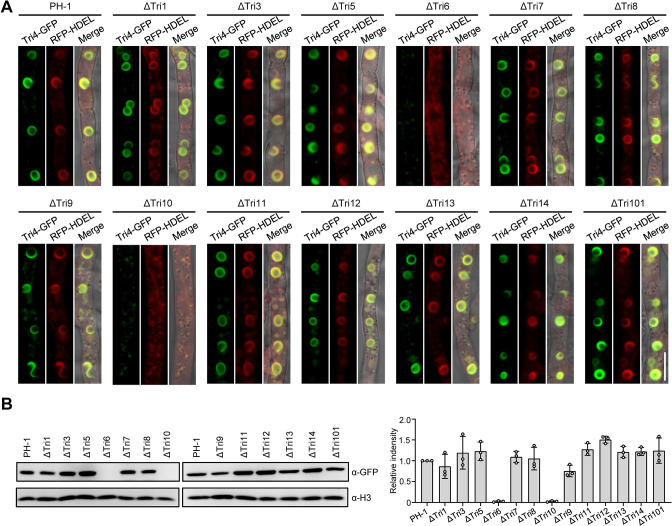
Regulation of the Tri4-marked TSS formation by the *TRI* cluster. (**A**) The effect of gene deletion in the *TRI* cluster on the formation of TSS under DON inducing conditions. The wild-type strain and *TRI* gene deletion mutants were grown in TBI medium at 28°C for 48 h. RFP-HDEL is used as the ER marker. Bar = 10 μm. (**B**) Accumulation of Tri4-GFP proteins in *TRI* gene deletion mutants was determined by western blot with the anti-GFP antibody (left panel). The protein abundance of H3 in each sample served as a loading control. The relative intensity of Tri4-GFP in each strain was shown in right panel, and was calculated by determining the intensity of Tri4-GFP band normalized to the intensity of H3 band. The relative intensity of the wild-type strain PH-1 was normalized to 1. Data represent the mean ± s.d. from three independent experiments.

Next, we attempted to explore the regulators for TSS formation. We first focused on the effect of the *TRI* cluster on the formation of toxisome since many fungal secondary metabolite (SM) biosynthesis gene clusters generally possess a self-regulation role. The *TRI* cluster contains 15 *TRI* genes, which are located at three different loci on different chromosomes in *F*. *graminearum*. Among them, the *TRI16* is only functional in *F*. *sporotrichioides* which produces T-2 toxin (a type of trichothecenes), while in *F*. *graminearum* the *TRI16* homologue is nonfunctional due to multiple insertions and deletions in its coding region [12]. Thus the remaining 13 *TRI* genes were knocked out individually in the np-Tri4-GFP/RFP-HDEL dual labeled strain to determine the function of *TRI* cluster on toxisome-shaped structure formation. Since *TRI* gene cluster hardly expressed under normal nutrient-rich medium, TSS formation in mycelia of these *TRI* gene deletion mutants harboring the tagged Tri4-GFP/RFP-HDEL was examined after incubation of each strain in TBI medium. All the *TRI* gene deletion mutants except for *TRI6* and *TRI10* produced typical TSS, and the Tri4-GFP labeled TSS co-localized with RFP-HDEL labeled ER (Fig 5A). In the ΔTri6 and ΔTri10 mutants, the Tri4-GFP fluorescent signals decreased dramatically and no typical TSS were observed in the mycelia, while the RFP-HDEL labeled ER was also faint (Fig 5A). Both *TRI6* and *TRI10* have been identified as positive regulatory genes in the *TRI* cluster for regulating the expression of trichothecene biosynthetic enzyme-encoding genes [15]. Consistently, the amounts of Tri4-GFP protein in ΔTri6 and Δtri10 mutants were significantly reduced or undetectable as compared with that in the wild-type strain after 48 h induction in TBI medium (Fig 5B). Whereas, except for *TRI6* and *TRI10*, the protein levels of Tri4-GFP in the remaining *TRI* gene deletion mutants were similar to or slightly higher than that in the wild-type strain (Fig 5B). These results indicate that in the *TRI* cluster, *TRI6* and *TRI10* are essential for the Tri4-marked TSS formation, which may result from the significantly decreased amount of Tri4 protein in these two gene deletion mutants.

### The UPR signaling pathway is not involved in TSS formation in *F*. *graminearum*

During formation of toxisome in TBI medium, *F*. *graminearum* cells are subjected to many stress conditions, such as nutrient deprivation, acidic pH, reactive oxygen species (ROS), which are potential inducers provoking ER stress [34,35]. In addition, ER-localized Tri proteins (Tri4 and Tri1) were highly induced during DON induction [4,25], which might also result in ER stress. To mitigate ER stress, eukaryotic cells activate a signal transduction pathway called the UPR, which maintains ER homeostasis by regulating the expression of numerous genes encoding ER chaperones, folding enzymes and other proteins [34]. In *Saccharomyces cerevisiae*, the UPR pathway is composed of the kinase Ire1 and the bZIP transcription factor Hac1. Upon ER stress, Ire1 is activated and then removes the unconventional intron of the *HAC1* mRNA via its endoribonuclease domain in a spliceosome-independent manner. Spliced *HAC1* mRNA is subsequently translated to produce an active Hac1 protein to upregulate expression of UPR target genes [34]. To determine whether the UPR signaling pathway contributes to *F*. *graminearum* toxisome formation, we first tried to knock out the homologue of Ire1 (FGRAMPH1_01G18735) and Hac1 (FGRAMPH1_01G11295) in *F*. *graminearum*. However, this attempt failed after screening over 100 ectopic transformants from at least three independent transformation experiments, indicating that these two genes are likely to be essential in *F*. *graminearum* due to high homologous recombination efficiency in this fungus [36,37].

**Fig 6:**
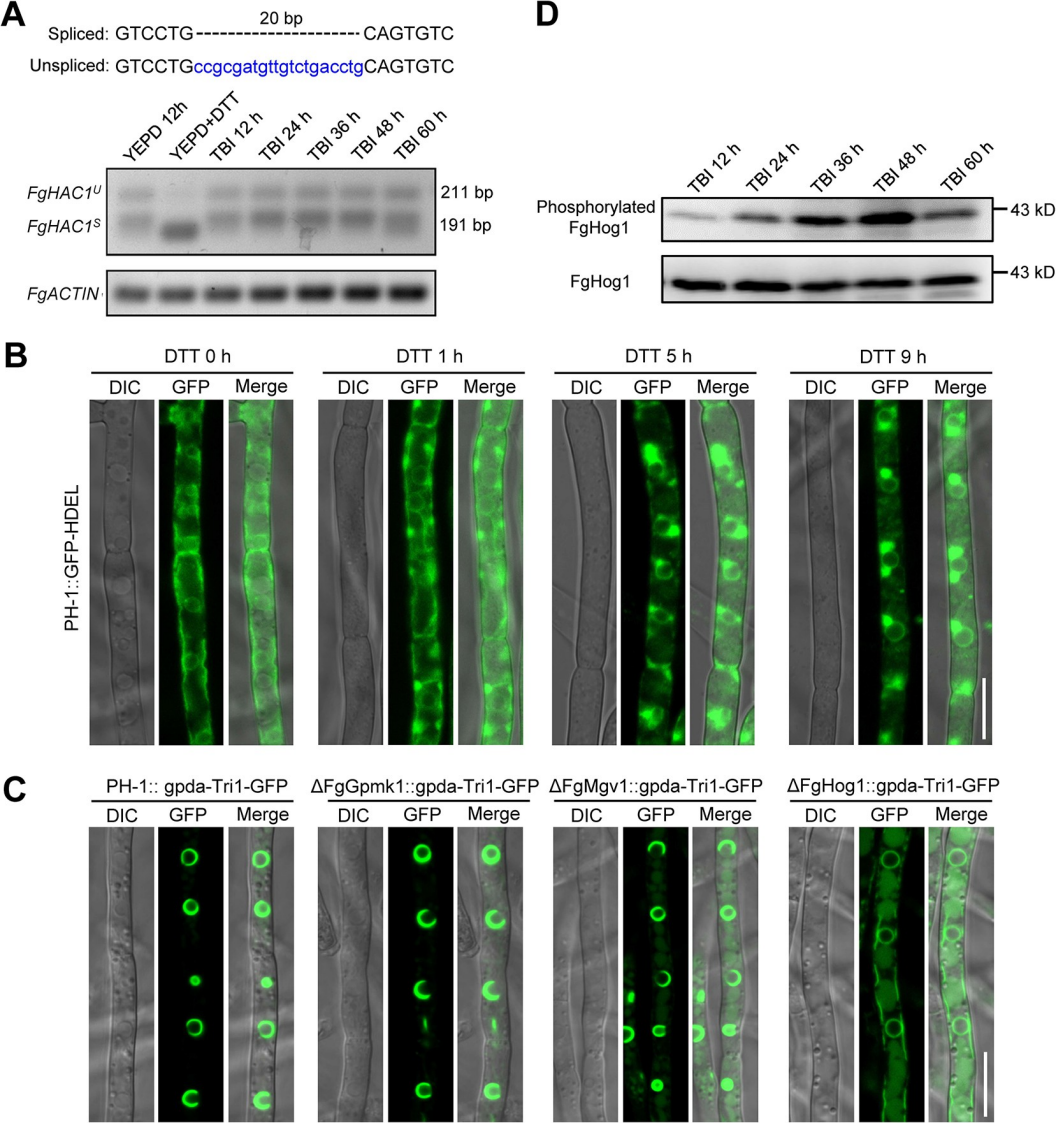
The HOG, but not the UPR signaling pathway, is involved in TSS assembly in *F*. *graminearum*. (**A**) The *FgHAC1* mRNA splicing pattern in TBI and YEPD medium. DNA sequence alignment of the spliced and unspliced *FgHAC1* (upper panel). The 20 bp non-canonical intron is indicated with lowercase blue letters (upper panel). The *FgHAC1* mRNA splicing pattern was analyzed by RT-PCR using the total RNA of PH-1 after incubation for the indicated times in TBI and YEPD medium (lower panel). PH-1 cultured in YEPD for 12 h and then treated with the ER stress agent DTT (10 mM) for 2 h was used as the positive control. Unspliced and spliced *FgHAC1* transcripts yielded 211 and 191-bp products, respectively. The transcripts of *FgACTIN* gene (FGRAMPH1_01G24551) served as the loading control. (**B**) DTT induces an aggregation of ER membrane. The strain PH-1::GFP-HDEL was used to visualize the ER and cultured in YEPD medium for 12 h followed by treatment with DTT for the indicated times. Bar = 10 μm. (**C**) The FgHog1 deletion mutant failed to induce the TSS formation under DON non-inducing conditions (YEPD medium). Images of each strain were taken after incubation in YEPD at 28°C for 48 h. Bar = 10 μm. (**D**) Time course analysis of the phosphorylation of FgHog1 in TBI. After growing in TBI medium at 28°C for the indicated times, mycelia of PH-1 were harvested for protein extraction in western blot analysis. FgHog1 and phosphorylated FgHog1 were detected using anti-Hog1 antibody and Anti-phospho-p38 antibody, respectively.

*F*. *graminearum HAC1* was predicted to contain a 20-nt non-canonical intron spliced by Ire1 [38], with a conserved Ire1 cleavage motif CNG’CNGN at the 5’ and 3’ boundary of the intron (Fig 6A, upper panel). To further investigate whether the UPR pathway regulates toxisome formation, we checked the *FgHAC1* mRNA splicing pattern under DON inducing (TBI medium) and non-inducing (YEPD medium) conditions with RT-PCR. The YEPD medium supplemented with dithiothreitol (DTT), an ER stress agent, was used as a positive control. Under normal nutrient conditions (YEPD), two bands: one unspliced (*FgHAC1*^*U*^) and the other spliced (*FgHAC1*^*S*^), were detected (Fig 6A, lower panel), indicating that even without an ER-stressing stimulus, the UPR pathway was partially activated. Upon ER stress (YEPD+DTT), the splicing of *FgHAC1* was triggered intensively, indicating a significant activation of the UPR signaling pathway under DTT treatment (Fig 6A, lower panel). DNA sequencing of the two bands confirmed that the 20-nt non-canonical intron (Fig 6A, upper panel) was excised in the spliced band. Unexpectedly, the splicing band patterns of *FgHAC1* in TBI conditions during the time course of 0–60 hours were similar to that in YEPD medium (Fig 6A, lower panel). Therefore, it is reasonable to speculate that the UPR signaling pathway is not further activated under DON inducing condition as comparison to that under the DON non-inducing condition.

Given that the ER stress inducer DTT is able to intensively activate the UPR pathway in *F*. *graminearum* (Fig 6A), we wondered whether the ER would be reorganized with DTT treatment, a condition inducing the UPR pathway. Using the ER marker GFP-HDEL, we found that DTT treatment induced globular aggregation structures linked with the perinuclear- and peripheral ER, which was more apparent with prolong treatment time (Fig 6B). However, this ER remodeling mediated by DTT was clearly distinct from TSS, with strongly thickened spherical and crescent structures around nuclear (Figs 1A, 1B, 3A and 3B). These results suggest that the UPR signaling pathway is not required for the toxisome formation in *F*. *graminearum*.

### The HOG signaling pathway is important for TSS formation

To further identify potential signal pathways that are involved in TSS formation, we focused on the three major mitogen-activated protein kinase (MAPK) signal transduction pathways: the cell wall integrity (CWI) signaling pathway, the high osmolarity glycerol (HOG) pathway, and the penetration pathway, which have all been reported to regulate *F*. *graminearum* DON production and expression of the *TRI* genes [4]. Three central components of these pathways: FgGpmk1 (MAPK of the penetration pathway), FgMgv1 (MAPK of the CWI pathway), and FgHog1 (MAPK of the HOG pathway) were thus knocked out in the strain PH-1::gpda-Tri1-GFP. We used this strain due to that the expression of Tri1-GFP protein was driven by the gpda promoter, which could maintain at a relatively stable level to avoid the effect of disruption of these kinases on the expression of *TRI1-GFP* gene. Results showed that the FgGpmk1 and FgMgv1 deletion mutants produced normal TSS as the wild-type strain in YEPD medium, whereas the FgHog1 deletion mutant failed to form the typical toxisome-shaped structures (Fig 6C), suggesting that the HOG signaling pathway is required for the ER remodeling into TSS.

To further verify that the HOG pathway is involved in TSS formation, we examined the phosphorylation pattern of FgHog1 (a marker for the activation of the HOG pathway) under DON inducing conditions. During the time course of DON induction in TBI medium, phosphorylation level of FgHog1 continuously increased between 12–48 hours, reached peak level at 48 h, and then decreased at 60 h (Fig 6D). This trend of phosphorylation of FgHog1 during DON induction highly correlated with the formation of toxisome structure, which also reached its peak at 48 h under DON inducing conditions [25]. These results indicate that the HOG pathway is involved in regulating toxisome formation during DON induction in *F*. *graminearum*.

### A novel chemical ZJU212 blocks toxisome formation and DON biosynthesis

**Fig 7:**
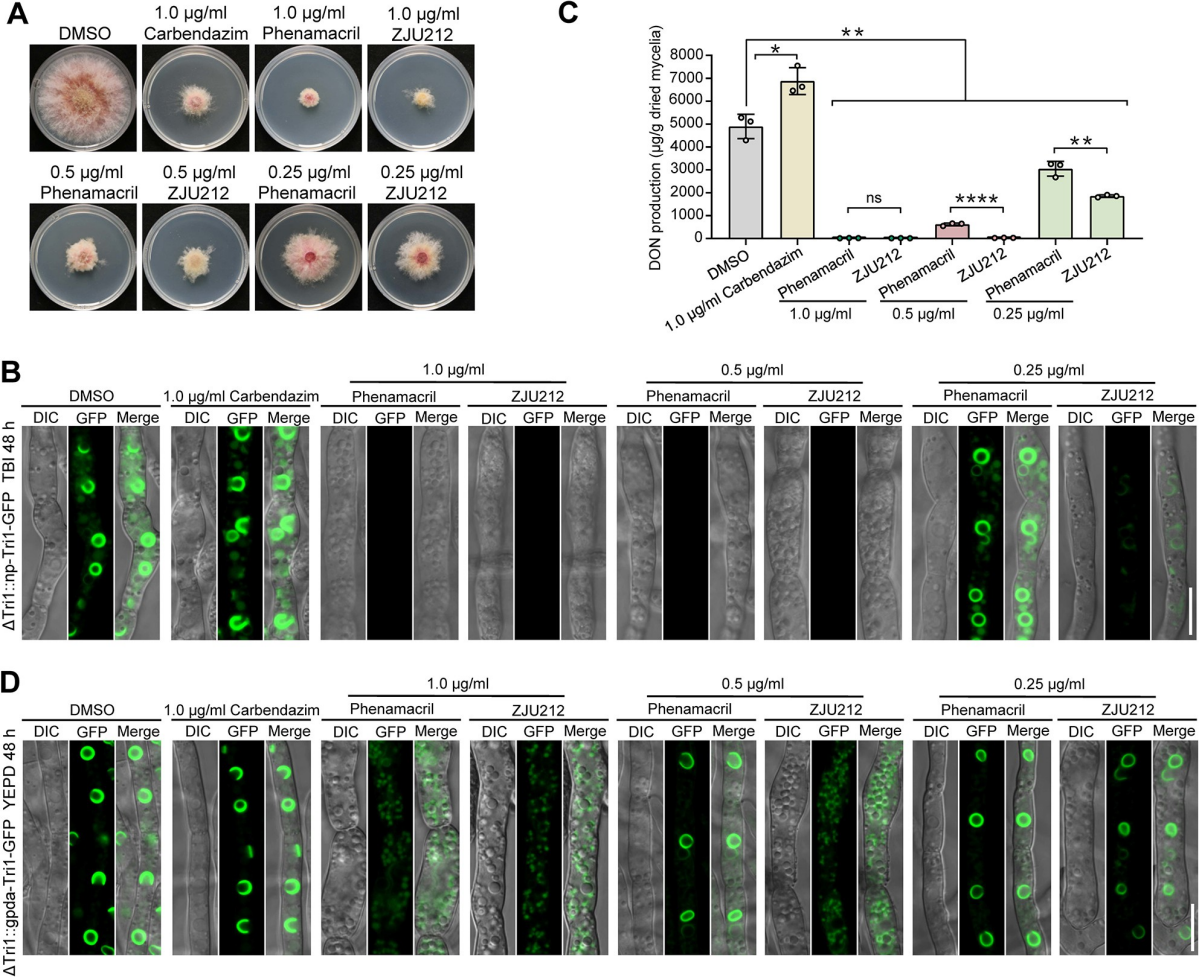
The novel compound ZJU212 effectively blocks the ER remodeling into TSS and subsequent DON biosynthesis. (**A**) Inhibition of each compound at the indicated concentration against mycelial growth of *F*. *graminearum* on PDA. DMSO was used as a control. Photos were taken after 2 days of incubation at 25°C on PDA amended without or with relevant compounds. (**B**) Toxisome formation in the mycelia of ΔTri1::np-Tri1-GFP (under native promoter) treated with each antifungal compound at indicated concentrations. After growth in TBI for 24 h, ΔTri1::np-Tri1-GFP was treated with antifungal compounds at indicated concentrations for another 24 h before examination. Bar = 10 μm. (**C**) DON production was assayed for the wild-type PH-1 grown in TBI supplemented with each antifungal compound at indicated concentrations. DON was extracted from mycelia of each strain cultured in TBI for 7 days and each compound was added after incubation of PH-1 in TBI medium for 24 h. Data represent the mean ± s.d. from three independent experiments. Statistical differences were analyzed by two-sided, unpaired Student’s *t*-test (ns: not significant, *P < 0.05, **P < 0.01, ****P < 0.0001). (**D**) TSS formation in the mycelia of ΔTri1::gpda-Tri1-GFP (under gpda promoter) treated with each antifungal compound at indicated concentrations. After growth in YEPD medium for 12 h, ΔTri1::gpda-Tri1-GFP was treated with antifungal compounds at indicated concentrations for another 36 h before examination. Bar = 10 μm.

Given that toxisomes are critical for DON biosynthesis, we are interested in screening compounds that could restrain toxisome formation, thereby inhibiting DON production. By using the "toxisome formation inhibitor screening" assay (see Materials and Methods), active compounds that effectively restrict toxisome formation were screened. Briefly, the reporter strain ΔTri1::np-Tri1-GFP was cultured in 24-wells plates supplemented with TBI medium for 24 h. Subsequently, individual tested compound was added to each well, incubated for another 24 h, and then fluorescent signal was measured. A total of 70 derivatives of phenamacril, a commercial fungicide which has been showed to target the myosin I of *F*. *graminearum* and subsequently inhibited toxisome formation [25], were designed and tested for their activity against toxisome formation. After two rounds of screening, a novel phenamacril derivative ZJU212 (S5 Fig), with a chemical name of Ethyl (Z)-3-amino-2-cyano-3-(4-(1,2,2-trimethylhydrazinyl) phenyl) acrylate was found to be very effective against mycelial growth and toxisome formation (Fig 7A and 7B). As shown in Fig 7B, under DON inducing conditions, no Tri1-GFP fluorescent signals and toxisomes were observed in the mycelia treated with 1.0 or 0.5 μg/ml ZJU212 or phenamacril, while intense fluorescence signals and typical toxisomes were measured in non-treated and carbendazim-treated (another commercial fungicide) mycelia. When 0.25 μg/ml of phenamacril and ZJU212 was used, intense fluorescence signals and toxisome structures like the non-treatment control were observed in phenamacril-treated mycelia, while Tri1-GFP signals decreased noticeably and only faint ER structures was observed in ZJU212-treated mycelia (Fig 7B). Correspondingly, DON production of *F*. *graminearum* wild-type strain treated with 1.0 μg/ml phenamacril and ZJU212 decreased dramatically, while treatment with 1.0 μg/ml carbendazim even stimulated DON biosynthesis (Fig 7C). However, the inhibitory activity of ZJU212 against DON synthesis is significantly higher than that of phenamacril at a concentration of 0.5 or 0.25 μg/ml (Fig 7C). These results indicate that ZJU212 can inhibit toxisome formation and DON biosynthesis more effectively than that of phenamacril.

Since treatment with ZJU212 and phenamacril under DON inducing conditions resulted in significantly reduced Tri1-GFP protein (Fig 7B), we still could not deduce whether these two compounds interfere with toxisome formation via the processes of ER remodeling or Tri gene transcription. To address this, the ΔTri1::gpda-Tri1-GFP strain was selected for further investigation. As shown in Fig 7D, no typical toxisome-shaped structures could be observed after treatment with ZJU212 and phenamacril at the concentration of 1.0 μg/ml, and Tri1-GFP signals were diffused in the cytoplasm. In contrast, 1.0 μg/ml carbendazim treatment was unable to block TSS formation (Fig 7D). However, when 0.5 μg/ml phenamacril and ZJU212 were used, typical TSS were observed in the mycelia treated with phenamacril, whereas ZJU212 still completely inhibited TSS formation (Fig 7D) although both phenamacril and ZJU212 at 0.25 μg/ml could not block the TSS formation (Fig 7D). These results indicate that the novel compound ZJU212 is more effective in blocking the remodeling of ER into TSS than that of phenamacril.

## Discussion

Many fungal SM biosynthetic pathways are compartmentalized to organelles to promote the efficiency of SM biosynthesis and serve as a self-protection system against self-toxicity of the SMs [4,14]. In the mycotoxin-producing fungus *F*. *graminearum*, the DON biosynthetic enzymes Tri1 and Tri4 are delivered to the specific cellular compartment named DON toxisome, which is a thickened spherical organelle derived from reorganized perinuclear and peripheral ER during DON sythesis [24]. In this study, we further demonstrated that the essence of toxisome-shaped structure (TSS) formation in *F*. *graminearum* is a response to the overproduction of ER-localized DON biosynthetic enzymes, including Tri1 and Tri4 as well as other ER-localized proteins (such as FgHmr1 and FgCnx). Overexpression of these proteins could lead ER to be reorganized into TSS even under DON non-inducing conditions (such as YEPD, CM and PDB medium) (S2 Fig). In addition, heterologous overexpression of Tri1- or Tri4-GFP in non-DON-producing fungi *F*. *oxysporum* f. sp. *lycopersici* and *F*. *fujikuroi* also led to TSS formation under DON non-inducing conditions (Fig 4A and 4B). Furthermore, homologous overproduction of the endogenous ER-resident protein Hmr1 in these two fungi dramatically remodeled the ER morphology from thin reticular structure to TSS (Fig 4C and 4D). These results strongly suggest that overproduction of ER-localized proteins could trigger the remodeling of ER into TSS in both DON producing and non-producing fungi.

It has been demonstrated that toxisome structure is an organized smooth ER (OSER) [24], which is a drastic remodeling in ER morphology resulting from smooth ER proliferations [39]. Previous studies have reported various OSER structures in yeast [40–42], mammalian [39,43–45], and plant cells [46,47], that can be induced by overexpression of some resident ER transmembrane proteins including HMG-CoA reductase (HMGR) [42,46,48], Sec61 [39], and cytochromes P450 or b5 [39,41,49]. In *Arabidopsis thaliana* and *Nicotiana benthamiana*, the OSER structure induced by HMGR-GFP overexpression appeared with hypertrophied aggregates near the nucleus as observed by confocal microscopy [46], which is different from OSER structures induced by HMGR in yeast [42] and in *Fusarium*. The latter was shown to be as thickened spherical, crescent and ovoid structures at perinuclear and peripheral positions (Figs 3A and 4D). Moreover, animal cells [39,50] and plant cells [46] can additionally exhibit a structure of OSER with cubic or hexagonal symmetry sinusoidal arrays, which have not been observed in yeast and in *F*. *graminearum* [24]. Thus, although OSER formation is likely a conserved response to elevated levels of specific ER-resident proteins among eukaryotes, the morphology of the ER remodeling related to OSER varies dramatically among different types of cells. Interestingly, it was previously noticed that not all ER-resident membrane proteins can induce OSER structure formation, even when expressed at high levels. For example, overexpression of the ER-localized stearoyl-CoA 9-desaturase Ole1 in yeast [42], the ER membrane protein Climp63 in HEK293 cells [51], or ER-localized protein P450 1A1 in the human embryonic kidney cell line 293 [49], could not cause ER remodeling into OSER structure. Similarly, in our study, overexpression of the ER-localized membrane protein FgCyp51A in *F*. *graminearum* failed to induce the OSER structure formation, although protein levels of FgCyp51A (indicated by GFP fluorescence intensity) have increased greatly compared to those under native promoter (S4 Fig). Therefore, only certain particular ER resident proteins can induce OSER morphogenesis, which is worth further investigation.

The expression level of the OSER-triggering proteins (*e*.*g*. some ER-localized proteins) is a key factor to induce native ER reorganization into OSER, as low quantities of these proteins failed to consistently produce OSER [52]. When cytochrome b5 in COS-7 cells was highly expressed, it induced OSER structures, but at lower expression, the ER network was not disturbed [39]. Consistently, in our study, we found that endogenous ER membrane proteins FgHmr1- and FgCnx-GFP driven by their native promoters (np) were expressed with low protein levels and localized to thin reticulate ER, while overexpression of these proteins by gpda promoter led ER to be remodeled into TSS (Fig 3). The amount of FgCnx-GFP protein increased about 7 folds under the strong gpda promoter as compared to that under the np (Fig 3D), whereas the protein level of FgHmr1-GFP increased for more times since compared with the gpda-FgHmr1-GFP, the np-FgHmr1-GFP band was almost undetectable (Fig 3C). Previous work showed that the protein level of Hmgr1 (ortholog of *Fusarium* FgHmr1) in *A*. *thaliana* elevated for 3 folds was sufficient for inducing OSER morphogenesis [46]. The compactin-resistant Chinese hamster cell exhibited extensive OSER structure, and expression of Hmgr1 in this cell line has been increased by 500-fold relative to the normal level [53]. Snapp et al. found that the OSER-inducing ER protein Sec61γ in COS-7 cells was accumulated three to nine times higher protein levels relative to the cells that had no OSER structures [39]. However, to date, there is not a universal standard to determine that to what extent is the overexpression level of a particular ER protein needed for inducing OSER structure formation.

To date, mechanisms of how cells regulate the biogenesis of OSER-related ER remodeling are largely unknown. In this study, we investigated possible pathways that are involved in regulation of TSS formation, and found that the UPR signaling pathway mediated by Ire1-Hac1 is not responsible for TSS formation in *F*. *graminearum*. In line with our results, the UPR pathway in *S*. *cerevisiae* did not contribute to karmellae (an OSER structure) triggered by the overexpression of ER-resident protein HMGR [54]. Moreover, the yeast Ire1 deletion mutant could assemble normal karmellaes that were structurally identical to those of wild-type cells [54]. Consistently, OSER structures induced by overproduction of another ER-resident protein cytochrome P450 were similar in both the wild-type and *IRE1*-deletion strains of yeast [55]. These results suggest that the Ire1-Hac1-mediated UPR pathway may be not involved in OSER biogenesis in yeast and *F*. *graminearum*, likely the same case in other fungal species.

It is worth mentioning that deletion of the central kinase FgHog1 of HOG pathway resulted in defect of TSS formation in *F*. *graminearum* (Fig 6C). Previous studies have suggested that OSER formation is accompanied by an increase of membrane lipid components [49,56–57], and disruption of phospholipid biosynthesis gene affects the formation of HMGR-induced OSER structures in *S*. *cerevisiae* [42]. Therefore, it is rational to speculate that there might be a great demand for biosynthesis of membrane lipid components, such as phospholipids and ergosterol during toxisome formation in *F*. *graminearum*. A recent study in our lab established that the HOG pathway positively regulates the transcription of genes in ergosterol biosynthesis pathway through phosphorylating (activating) a novel transcription factor FgSR, which directly binds to the promoters of a set of ergosterol biosynthesis genes [58]. Therefore, ergosterol biosynthesis regulated by the FgHog1-FgSR cascade might provide an important lipid stock for toxisome formation in *F*. *graminearum*. Consistent with this hypothesis, although FgSR can be deleted in the wild-type strain, we failed to obtain a FgSR deletion mutant in the Tri1-overexpression strain PH-1::gpda-Tri1-GFP in this study, implying the importance of FgHog1-FgSR cascade in ER remodeling. Collectively, these results suggest that the HOG signaling pathway is required for the Tri1-overexpression regulated TSS formation likely owing to its ability to regulate sterol synthesis in *F*. *graminearum*.

Due to lack of highly resistant wheat cultivars, application of fungicides is still an effective strategy for management of FHB and DON contamination on wheat. However, several studies have reported that some commercial fungicides at sub-lethal concentrations, such as epoxyconazole, propiconazole, tebuconazole, and fluquinconazole, could stimulate DON biosynthesis, although their applications were able to reduce disease significantly [59,60]. In current study, we found that Tri4-marked toxisome structures were abolished by disrupting the transmembrane domain of Tri4 (Fig 2B), and the strain ΔTri4::Tri4^ΔTMD^-GFP containing the catalytic domain alone was almost unable to produce DON (Fig 2D), further supporting the critical role of the toxisome for DON biosynthesis. Since mycotoxin DON is a key virulence factor for *F*. *graminearum* infection on wheat tissue, toxisome would be a potential biomarker for screening antifungal compounds against FHB and DON biosynthesis. In a previous study, we found that the myosin I of *F*. *graminearum* (FgMyo1) plays critical roles in DON toxisome formation and DON biosynthesis via interacting with Tri1, and inhibition of myosin I by the small molecule phenamacril led to marked reduction in DON biosynthesis [25]. Using toxisome labeled with Tri1-GFP as a biomarker, we discovered that the novel compound ZJU212 exhibited much higher activity against toxisome formation and DON biosynthesis than that of phenamacril (Fig 7B–7D). Similar to phenamacril, ZJU212 has a low or even no activity against mycelial growth of other fungal species including *Botrytis cinerea*, *Magnaporthe oryzae* and *S*. *cerevisiae* (S6 Fig). In addition, ZJU212 did not affect wheat seed germination and the growth of wheat seedlings even at a high concentration (10 μg/mL) (S7 Fig), indicating this compound is quite safe for many other eukaryotes. Furthermore, although the inhibitory activity of ZJU212 against *F*. *graminearum* is similar to that of phenamacril, the inhibitory activity of ZJU212 against other *Fusarium* genus fungi including *F*. *oxysporum* f. sp. *lycopersici*, *F*. *verticillioides*, and *F*. *fujikuroi* is significantly better than that of phenamacril (S6 Fig). Thus, this novel compound ZJU212 may have a high potential to be developed as a fungicide for the management of *Fusarium* disease and DON contamination.

In conclusion, the ER remodeling into toxisome structure plays an important role in *F*. *graminearum* DON biosynthesis, and the essence of ER remodeling into toxisome upon DON induction is a response to the overproduction of ER-localized DON biosynthetic enzymes. Therefore, blocking the ER remodeling would provide a new avenue for fungicide development for the management of FHB and DON contamination.

## Materials and methods

### Fungal strains and growth conditions

The *F*. *graminearum* wild-type strain PH-1 (NRRL 31084) was used as a parental strain for transformation experiments. The wild-type strain of *F*. *oxysporum* f. sp. *lycopersici* (*Fol*) was isolated from tomato plants affected by wilt disease in Hunan province, China, and *F*. *fujikuroi* wild-type strain was isolated from a rice seedling suffering from rice bakanae disease in Jiangsu province, China. The wild-type strains and resultant transgenic strains were cultrured on PDA (200 g potato, 20 g glucose, 10 g agar and 1 L water) at 25°C for mycelial growth. The carboxymethyl cellulose (CMC) liquid medium (1 g NH_4_NO_3_, 1 g KH_2_PO_3_, 0.5 g MgSO_4_·7H_2_O, 1 g yeast extract, 15 g CMC and 1 L water) was used for fungal conidiation. Fungal strains were grown on YEPD liquid medium (10 g peptone, 3 g yeast extract, 20 g D-glucose and 1 L water, pH 7.0) for harvesting vegetative hyphae. For trichothecene induction, each strain was grown in liquid TBI medium (30g sucrose, 1g KH_2_PO_4_, 0.5g MgSO_4_. 7 H_2_O, 0.5g KCl, 0.01g FeSO_4_. 7 H_2_O, 1.47g putrescine hydrochloride, trace elements and 1 L water, pH 4.5) [61].

### Construction of gene deletion mutants

Targeted gene deletion was generated using the double-joint (DJ) PCR method [62]. First, the 5’ and 3’ flanking regions of the target gene were amplified from genomic DNA of the wild type using primers listed in S1 Table. The flanking fragments were then fused with the hygromycin-resistance gene cassette (*HPH*) with overlapping PCR, and the resulting fusion fragment was transformed into the parental strain to replace the open reading frame (ORF) of the targeted gene by homologous recombination (S8 Fig showes an example of generating the *TRI1* deletion cassette). The transformation of *F*. *graminearum* strains was carried out via polyethyleneglycol (PEG) mediated protoplast transformation method, as described previously [63,64]. *F*. *graminearum* protoplasts were isolated by treating fresh mycelia with 30 mg driselase (D9515, Sigma, St.Louis, MO), 200 mg lysozyme (RM1027, RYON, Shanghai, China), and 200 mg cellulose (RM1030, RYON, Shanghai, China) in 10 ml 0.7 M NaCl, and then incubated at 30°C with agitation (85 rpm) for 4 h. Putative targeted gene deletion mutants were identified by PCR (S8 Fig) with primer pairs listed in S1 Table. Hygromycin B was added to a final concentration of 100 μg ml^-1^ for hygromycin resistant transformant selection.

### Generation of fluorescence-labeled strains

To construct the Tri4-GFP fusion cassette under native promoter, the open reading frame (ORF) of Tri4 (without the stop codon) together with its native promoter region was amplified with primers listed in S1 Table. The resulting PCR products were co-transformed with Xho1-digested pYF11 [65] into the yeast strain XK1-25. Subsequently, the Tri4-GFP fusion vector was recovered from yeast transformants and then transferred into *E*. *coli* strain DH5α for amplification. The final Tri4-GFP fusion construct was verified by sequencing to obtain the correctly conjugated expression vector. Tri1-GFP, Tri4^ΔTMD^-GFP and Tri4^Δp450^-GFP fusion cassettes driven by their native promoters were constructed using the same strategy. To construct the Tri4-GFP and Tri1-GFP fusion cassettes under the strong constitutive gpda promoter [66,67], the ORF of Tri4 or Tri1 (without the stop codon) was amplified and then fused with the gpda promoter with overlapping PCR. The resulting gpda-Tri4 or gpda-Tri1 fusion fragment was co-transformed with Xho1-digested pYF11 into the yeast strain XK1-25 to obtain gpda-Tri4-GFP or gpda-Tri1-GFP fusion vector and subsequently transferred into DH5α for amplification. The primer pairs used for vector construction are listed in S1 Table.

To create the RFP-HDEL construct, the overlapping PCR-based method was adopted. Briefly, the RP27 promoter in pYF11, ER targeting signal peptide sequence from *F*. *graminearum* BiP protein (gene locus FGSG_09471, 1–33 aa in N-terminus), RFP sequence ending with HDEL ER retention signal (CACGACGAGTTG), and nourseothricin-resistance gene cassette (*NAT1*) were amplified and fused with overlapping PCR to generate the RP27-BiP-RFP-HDEL-NAT1 fusion fragment. The resulting fusion fragment was transformed into the wild-type strain PH-1 for ectopic insertion into the genome. Nourseothricin resistant transformants were isolated with PDA supplemented with 50 μg ml^-1^ nourseothricin.

Since FgHmr1 (HMG-CoA reductase) is essential in *F*. *graminearum*, the FgHmr1-GFP fusion cassette under the native promoter was constructed using a knock-in strategy as described previously [26] to avoid transferring extra FgHmr1 copy into the *F*. *graminearum* strain. In brief, the upstream and downstream regions (up and down) flanking the FgHmr1 stop codon, the GFP sequence, and geneticin (G418) resistance cassette were amplified and fused with overlapping PCR to generate the up-GFP-G418-down fragment. The resulting fusion fragment was transformed into the wild-type strain PH-1 to replace the stop codon of FgHmr1 by homologous recombination (S9A Fig). Geneticin resistant transformants were selected with geneticin at a final concentration of 100 μg ml^-1^. The resultant strain np-FgHmr1-GFP was identified by PCR (S9B Fig). The np-FgCnx-GFP, np-FolHmr1-GFP and np-FfHmr1-GFP strains driven by native promoters were constructed using the same strategy. To construct the FgHmr1-GFP fusion cassette under the gpda promoter, the hygromycin-resistance gene cassette (*HPH*) and the gpda promoter were amplified and fused with overlapping PCR to generate the HPH-gpda fragment. The 5’ and 3’ flanking regions (up and down) of the native promoter of FgHmr1 were amplified and then fused to the HPH-gpda fragment (S9C Fig). The resulting up-HPH-gpda-down fragment was transformed into the strain np-FgHmr1-GFP to replace the native promoter of FgHmr1 and generate the gpda-FgHmr1-GFP strain (S9C Fig). The gpda-FgCnx-GFP, gpda-FolHmr1-GFP and gpda-FfHmr1-GFP strains driven by the gpda promoter were constructed using the same strategy. All strains were verified by PCR assay (S9D Fig) with relevant primers listed in S1 Table.

### Microscopic observations for toxisome formation

To observe the toxisome formation pattern in various fluorescence-labeled strains in Figs 1–7, 250 μl of conidial suspension (10^6^ conidia/ml) of each strain was inoculated into 25 ml TBI or YEPD liquid medium and incubated at 28°C for 48 h in a shaker (150 rpm) in total darkness. Fluorescent signals in the mycelia were observed under a Zeiss LSM880 confocal microscopy (Gottingen, Niedersachsen, Germany). The laser excitation wavelength was set at 488 nm for GFP (green fluorescence) and at 561 nm for RFP (red fluorescence). ZeissZEN 2012 software was used for image generation and analysis.

### Western blotting assay

To obtain fresh mycelia for protein extraction, 250 μl of conidial suspension (10^6^ conidia ml^−1^) of each strain was inoculated into 25 ml TBI or YEPD liquid medium and incubated at 28°C with agitation (150 rpm) in the dark for 48 h. Mycelia from each strain were then harvested, washed with deionized water and ground into fine powder in liquid nitrogen. Approximately 100 mg of fine ground mycelia were suspended with 1 ml protein lysis buffer (50 mM Tris-HCl [pH 7.5], 150 mM NaCl, 5 mM EDTA, 1% Triton X-100, 1:100 v/v protease inhibitor cocktail [Sangon Biotech, Shanghai, China]). After homogenization with a vortex shaker, the lysate was centrifuged at 15000 × *g* for 20 min at 4°C. Then, 100 μl of supernatant was mixed with 25 μl of 5 × loading buffer and boiled for 5 min. The resulting proteins were separated by 12% sodium dodecyl sulfate-polyacrylamide gel electrophoresis (SDS-PAGE) and transferred to Immobilon-Ptransfer membrane (Millipore, Billerica, MA, USA). GFP-tagged proteins were detected with monoclonal anti-GFP (ab32146, Abcam, Cambridge, UK, 1:10000 dilution) antibody. Samples were also detected with monoclonal anti-histone H3 antibody (M1306-4, HuaAn Biotechnology, Hangzhou, China, 1:10000 dilution) as a reference. Total protein of FgHog1 was detected with the anti-Hog1 antibody (sc-165,978, Santa Cruz, CA, USA; 1:2000 dilution), and the phosphorylated FgHog1 was detected with the anti-phospho-p38 antibody (#9211, Cell Signalling Technology, Boston, MA, USA; 1:2000 dilution) [58]. The experiments were repeated three times.

### DON production assay

To determine DON production of each strain in TBI medium in Figs 2 and 7, 100 μl of conidial suspension (10^6^ conidia ml^−1^) of each strain was inoculated into 25 ml TBI medium and incubated at 28°C for 7 days in a shaker (150 rpm) in the dark. DON production of each strain in TBI medium was quantified by using a competitive ELISA based DON detection plate kit Wis008 (Wise Science, Zhenjiang, China) according to the manufacturer’s instructions. The experiment was repeated three times.

To quantify DON production of each strain in YEPD and TBI medium in S1 Fig, 100 μl of conidial suspension (10^6^ conidia ml^−1^) of each strain was inoculated into 25 ml YEPD or TBI medium and incubated at 28°C for 7 days in a shaker (150 rpm) in the dark. The cell-free supernatant was filtered and passed through a SampliQ Amino (NH2) solid phase extraction column (Agilent Technologies), and 4 ml of the purified extract were evaporated to dryness under a nitrogen stream. The residue was dissolved in 1 ml methanol:water (40:60, v/v), followed by centrifugation at 10,000 rpm and subsequently analyzed by LC-MS. The experiment was conducted three times.

### RNA extraction and RT-PCR

Total RNA was isolated from fresh mycelia of each fungal sample with the TRIzol reagent (TaKaRa Biotechnology Co., Dalian, China) following the manufacturer’s instructions. For each sample, 1 μg of total RNA was reverse transcribed into first-strand cDNA using a HiScript II Q RT SuperMix kit (R223-01, Vazyme Biotech Co., Nanjing, China) according to the manufacturer’s instructions. To confirm the splicing pattern of *FgHAC1* mRNA under the UPR and DON inducing conditions, reverse transcription PCR (RT-PCR) reaction was performed using 2 × Taq Master Mix (P112-01, Vazyme Biotech Co., Nanjing, China), using primers specific listed in S1 Table. The PCR reaction was set at 94°C for 5 min, followed by 32 cycles of 94°C for 30 s, 55°C for 30 s, and 72°C for 12 s. The gene *FgACTIN* (FGRAMPH1_01G24551) was used as an internal control. The experiment was performed for three biological replications.

### Toxisome formation inhibitor screening assay

The strain ΔTri1::np-Tri1-GFP, in which Tri1-GFP was expressed under its native promoter in the ΔTri1 background, was used as the fluorescent reporter strain for screening assay. The TBI medium supplemented with conidia of the reporter strain (10^4^ conidia/ml at a final concentration) was added into the 24-well plate (2 ml/well). After static incubation at 28°C for 24 h, each compound to be tested (at 0.5 μg/ml) was added into a well and repeated for three wells. The solvent DMSO with the same volume was used as the non-treatment control. After incubation for another 24 h, the fluorescent intensity in each well was scanned with the Varioskan Flash Multimode Reader (Thermo Scientific, MA, USA) for the first round screening. The wells with lower fluorescent signals compared with that of the control treatment were further observed by a Zeiss LSM880 confocal microscopy (Gottingen, Niedersachsen, Germany) to confirm the inhibitory activity against toxisome formation. A total of 70 antifungal compounds were tested. The experiment was repeated three times.

## Supporting information

S1 Fig DON production of strains with native and gpda promoters in YEPD medium or TBI medium. Strains ΔTri4::np-Tri4-GFP::RFP-HDEL, ΔTri4::gpda-Tri4-GFP::RFP-HDEL, ΔTri1::np-Tri1-GFP::RFP-HDEL and ΔTri1::gpda-Tri1-GFP::RFP-HDEL in Fig 1 were incubated in YEPD or TBI medium for 7 d and determined for DON production by LC-MS. Data represent the mean ± s.d. from three independent experiments.
(TIF)

S2 Fig Formation of TSS in CM and PDB media. Toxisome-shaped structures were formed in the mycelia of strains ΔTri4::gpda-Tri4-GFP (A) and ΔTri1::gpda-Tri1-GFP (B) after 48 h incubation at 28°C in CM and PDB liquid media. Bar = 10 μm.
(TIF)

S3 Fig Localization of Tri4^ΔTMD^-GFP and Tri1-RFP in the dual-labelled strain ΔTri4::Tri4^ΔTMD^-GFP::Tri1-RFP. The dual-labelled strain was grown in TBI medium at 28°C for 48 h before observation. Bar = 10 μm.
(TIF)

S4 Fig Overexpression of the ER-resident protein FgCyp51A failed to trigger the ER remodeling into toxisome-shaped structure. Localization of FgCyp51A-GFP under native promoter (upper panels) and gpda promoter (lower panels) in the non-inducing YEPD medium. Each strain was grown in YEPD medium at 28°C for 48 h. Bar = 10 μm.
(TIF)

S5 Fig Chemical structure of the novel antifungl compound ZJU212. Chemical structures of the fungicide phenamacril (A) and its derivative ZJU212 (B).
(TIF)

S6 Fig Antifungal activity of ZJU212 and phenamacril against different fungi. *Fusarium graminearum*, *Fusarium oxysporum* f. sp. *lycopersici*, *Fusarium verticillioides*, *Fusarium fujikuroi*, *Botrytis cinerea*, *Magnaporthe oryzae*, and *Saccharomyces cerevisiae* were inoculated on PDA plates supplemented with various concentrations of ZJU212 and phenamacril as indicated. The inoculated plates were incubated at 25°C and imaged when mycelia on the control plate extended to the edge of plates. *S*. *cerevisiae* was grown on the YPDA plate amended with different concentrations of ZJU212 and phenamacril, and incubated at 30°C for 3 days before imaging. DMSO was used as a negative control.
(TIF)

S7 Fig Toxicity of ZJU212 to wheat seedlings and seed germination. (A) The effects of ZJU212 on wheat seedings. Wheat seeds were soaked in H_2_O supplemented with ZJU212 at a final concentration of 1, 5, and 10 μg/mL at 25°C for 24 h, then incubated for 7 days and photographed. The solvent DMSO was used as a non-treatment control. (B) The effects of ZJU212 on wheat seed germination. Wheat seeds were soaked in H_2_O supplemented with ZJU212 at the indicated concentration at 25°C for 24 h, and after another 3 days incubation, the seed germination percentage of each treatment was measured.
(TIF)

S8 Fig Schematic representation of gene disruption and validation of the transformants. (A) Schematic representation of the *TRI1* disruption strategy (left panel) and PCR assays for identification of *TRI1* gene deletion mutant (right panel). Binding positions of PCR primers (arrows indicated) used for the construction of *TRI1* deletion mutant are illustrated (left panel). Correct deletion was confirmed in two independent ΔTri1 transformants (#1–2) by PCR assay (right panel) using the primer pairs Tri1-id-F/Tri1-id-R indicated in left panel. Strain #2 was used for further studies. (B) Validation of *TRI* cluster gene deletion mutants in the parental strain PH-1::np-Tri4-GFP::RFP-HDEL by PCR assays. Correct deletion was confirmed in two or three independent deletion transformants by PCR assay using the primer pairs listed in S1 Table.
(TIF)

S9 Fig Scheme of constructs to generate Hmr1-GFP or Cnx-GFP fluorescence strains driven by native or gpda promoters. (A) Schematic representation of the knock-in strategy to generate the strain np-FgHmr1-GFP (expressing FgHmr1-GFP under the native promoter). By homologous recombination between flanking regions upstream (UP) and downstream (DOWN) of the stop codon of *FgHMR1* and homologous flanks of the UP-GFP-G418-DOWN construct, the stop codon is replaced by the GFP-G418 fusion fragment of the construct. The strains np-FgCnx-GFP, np-FolHmr1-GFP and np-FfHmr1-GFP driven by native promoters were constructed using the same strategy. (B) Identification of the correct transformants that expess FgHmr1-GFP under native promoter by using PCR primers as indicated in (A) (arrows). The strains np-FgCnx-GFP, np-FolHmr1-GFP and np-FfHmr1-GFP were also identified with primers listed in S1 Table. (C) Schematic representation of the strategy to generate the strain gpda-FgHmr1-GFP (expressing FgHmr1-GFP under the gpda promoter). By homologous recombination between the 5’ and 3’ flanking regions (UP and DOWN) of the native promoter of FgHmr1 in the strain np-FgHmr1-GFP and homologous flanks of the UP-HPH-gpda-DOWN construct, the native promoter is replaced by the HPH-gpda fusion fragment. The strains gpda-FgCnx-GFP, gpda-FolHmr1-GFP and gpda-FfHmr1-GFP driven by gpda promoters were constructed using the same strategy. (D) Identification of the correct transformants that expessing FgHmr1-GFP under gpda promoter by using PCR primers as indicated in (C) (arrows). The strains gpda-FgCnx-GFP, gpda-FolHmr1-GFP and gpda-FfHmr1-GFP were also identified with primers listed in S1 Table.
(TIF)

S1 Table A list of primers used in this study. (DOC)

S1 Data All numerical values used to generate graphs and histograms. Excel spreadsheet containing the underlying numerical data for Figs panels 2C, 2D, 5B, 7C, S1A, S1B and S7B.
(XLS)

## References

[1] Xu X, Nicholson P. Community ecology of fungal pathogens causing wheat head blight. Annu Rev Phytopathol. 2009; 47:83–103. doi: 10.1146/annurev-phyto-080508-081737 19385728

[2] Dean R, Van Kan JA, Pretorius ZA, Hammond-Kosack KE, Di Pietro A, Spanu PD, et al. The Top 10 fungal pathogens in molecular plant pathology. Mol Plant Pathol. 2012; 13(4):414–30. doi: 10.1111/j.1364-3703.2011.00783.x 22471698 PMC6638784

[3] Figueroa M, Hammond-Kosack KE, Solomon PS. A review of wheat diseases-a field perspective. Mol Plant Pathol. 2018; 19(6):1523–1536. doi: 10.1111/mpp.12618 29045052 PMC6638159

[4] Chen Y, Kistler HC, Ma Z. *Fusarium graminearum* Trichothecene Mycotoxins: Biosynthesis, Regulation, and Management. Annu Rev Phytopathol. 2019; 57:15–39. doi: 10.1146/annurev-phyto-082718-100318 30893009

[5] Ma Z, Xie Q, Li G, Jia H, Zhou J, Kong Z, et al. Germplasms, genetics and genomics for better control of disastrous wheat Fusarium head blight. Theor Appl Genet. 2020; 133(5):1541–1568. doi: 10.1007/s00122-019-03525-8 31900498

[6] Pestka JJ. Deoxynivalenol: mechanisms of action, human exposure, and toxicological relevance. Arch Toxicol. 2010; 84(9):663–79. doi: 10.1007/s00204-010-0579-8 20798930

[7] Arunachalam C, Doohan FM. Trichothecene toxicity in eukaryotes: cellular and molecular mechanisms in plants and animals. Toxicol Lett. 2013; 217(2):149–58. doi: 10.1016/j.toxlet.2012.12.003 23274714

[8] Bianchini A, Horsley R, Jack MM, Kobielush B, Ryu D, Tittlemier S, et al. DON occurrence in grains: A North American perspective. Cereal Foods World. 2015; 60:32–56. 10.1094/CFW-60-1-0032

[9] Varga E, Wiesenberger G, Hametner C, Ward TJ, Dong Y, Schöfbeck D, et al. New tricks of an old enemy: isolates of *Fusarium graminearum* produce a type A trichothecene mycotoxin. Environ Microbiol. 2015; 17(8):2588–600. doi: 10.1111/1462-2920.12718 25403493 PMC4950012

[10] Bai GH, Desjardins AE, Plattner RD. Deoxynivalenol-nonproducing *Fusarium graminearum* causes initial infection, but does not cause disease spread in wheat spikes. Mycopathologia. 2002; 153(2):91–8. doi: 10.1023/a:1014419323550 12000132

[11] Proctor RH, McCormick SP, Kim HS, Cardoza RE, Stanley AM, Lindo L, et al. Evolution of structural diversity of trichothecenes, a family of toxins produced by plant pathogenic and entomopathogenic fungi. PLoS Pathog. 2018; 14(4):e1006946. doi: 10.1371/journal.ppat.1006946 29649280 PMC5897003

[12] Alexander NJ, Proctor RH, Mccormick SP. Genes, gene clusters, and biosynthesis of trichothecenes and fumonisins in *Fusarium*. Toxin Rev. 2009; 28:198–215. 10.1080/15569540903092142

[13] Merhej J, Richard-Forget F, Barreau C. Regulation of trichothecene biosynthesis in *Fusarium*: recent advances and new insights. Appl Microbiol Biotechnol. 2011; 91(3):519–528. doi: 10.1007/s00253-011-3397-x 21691790

[14] Du L, Li S. Compartmentalized biosynthesis of fungal natural products. Curr Opin Biotechnol. 2021; 69:128–135. doi: 10.1016/j.copbio.2020.12.006 33450704

[15] Seong KY, Pasquali M, Zhou X, Song J, Hilburn K, McCormick S, et al. Global gene regulation by *Fusarium* transcription factors Tri6 and Tri10 reveals adaptations for toxin biosynthesis. Mol Microbiol. 2009; 72(2):354–67. doi: 10.1111/j.1365-2958.2009.06649.x 19320833

[16] Nasmith CG, Walkowiak S, Wang L, Leung WW, Gong Y, Johnston A, et al. Tri6 is a global transcription regulator in the phytopathogen *Fusarium graminearum*. PLoS Pathog. 2011; 7(9):e1002266. doi: 10.1371/journal.ppat.1002266 21980289 PMC3182926

[17] Menke J, Dong Y, Kistler HC. *Fusarium graminearum* Tri12p influences virulence to wheat and trichothecene accumulation. Mol Plant Microbe Interact. 2012; 25(11):1408–18. doi: 10.1094/MPMI-04-12-0081-R . 22835271

[18] Kistler HC, Broz K. Cellular compartmentalization of secondary metabolism. Front Microbiol. 2015; 6:68. doi: 10.3389/fmicb.2015.00068 25709603 PMC4321598

[19] Chanda A, Roze LV, Kang S, Artymovich KA, Hicks GR, Raikhel NV, et al. A key role for vesicles in fungal secondary metabolism. Proc Natl Acad Sci U S A. 2009; 106(46):19533–8. doi: 10.1073/pnas.0907416106 19889978 PMC2773199

[20] Chanda A, Roze LV, Linz JE. A possible role for exocytosis in aflatoxin export in *Aspergillus parasiticus*. Eukaryot Cell. 2010; 9(11):1724–7. doi: 10.1128/EC.00118-10 20870882 PMC2976301

[21] Roze LV, Chanda A, Linz JE. Compartmentalization and molecular traffic in secondary metabolism: a new understanding of established cellular processes. Fungal Genet Biol. 2011; 48(1):35–48. doi: 10.1016/j.fgb.2010.05.006 20519149 PMC2949687

[22] Fernández-Aguado M, Martín JF, Rodríguez-Castro R, García-Estrada C, Albillos SM, Teijeira F, et al. New insights into the isopenicillin N transport in *Penicillium chrysogenum*. Metab Eng. 2014; 22:89–103. doi: 10.1016/j.ymben.2014.01.004 24480587

[23] Upadhyay S, Xu X, Lowry D, Jackson JC, Roberson RW, Lin X. Subcellular compartmentalization and trafficking of the biosynthetic machinery for fungal melanin. Cell Rep. 2016; 14(11):2511–8. doi: 10.1016/j.celrep.2016.02.059 26972005 PMC4805463

[24] Boenisch MJ, Broz KL, Purvine SO, Chrisler WB, Nicora CD, Connolly LR, et al. Structural reorganization of the fungal endoplasmic reticulum upon induction of mycotoxin biosynthesis. Sci Rep. 2017; 7:44296. doi: 10.1038/srep44296 28287158 PMC5347122

[25] Tang G, Chen Y, Xu JR, Kistler HC, Ma Z. The fungal myosin I is essential for *Fusarium* toxisome formation. PLoS Pathog. 2018; 14(1):e1006827. doi: 10.1371/journal.ppat.1006827 29357387 PMC5794197

[26] Menke J, Weber J, Broz K, Kistler HC. Cellular development associated with induced mycotoxin synthesis in the filamentous fungus *Fusarium graminearum*. PLoS One. 2013; 8(5):e63077. doi: 10.1371/journal.pone.0063077 23667578 PMC3646755

[27] Blum M, Chang HY, Chuguransky S, Grego T, Kandasaamy S, Mitchell A, et al. The InterPro protein families and domains database: 20 years on. Nucleic Acids Res. 2021; 49:D344–D354. doi: 10.1093/nar/gkaa977 33156333 PMC7778928

[28] Burg JS, Espenshade PJ. Regulation of HMG-CoA reductase in mammals and yeast. Prog Lipid Res. 2011; 50(4):403–10. doi: 10.1016/j.plipres.2011.07.002 21801748 PMC3184313

[29] Williams DB. Beyond lectins: the calnexin/calreticulin chaperone system of the endoplasmic reticulum. J Cell Sci. 2006; 119:615–23. doi: 10.1242/jcs.02856 16467570

[30] Estrada P, Kim J, Coleman J, Walker L, Dunn B, Takizawa P, et al. Myo4p and She3p are required for cortical ER inheritance in *Saccharomyces cerevisiae*. J Cell Biol. 2003; 163(6):1255–66. doi: 10.1083/jcb.200304030 14691136 PMC2173705

[31] Flynn CM, Broz K, Jonkers W, Schmidt-Dannert C, Kistler HC. Expression of the *Fusarium graminearum* terpenome and involvement of the endoplasmic reticulum-derived toxisome. Fungal Genet Biol. 2019; 124:78–87. doi: 10.1016/j.fgb.2019.01.006 30664933 PMC6664814

[32] Liou AY, Molday LL, Wang J, Andersen JP, Molday RS. Identification and functional analyses of disease-associated P4-ATPase phospholipid flippase variants in red blood cells. J Biol Chem. 2019; 294(17):6809–6821. doi: 10.1074/jbc.RA118.007270 30850395 PMC6497962

[33] Long N, Xu X, Zeng Q, Sang H, Lu L. Erg4A and Erg4B are required for conidiation and azole resistance via regulation of ergosterol biosynthesis in *Aspergillus fumigatus*. Appl Environ Microbiol. 2017; 83(4):e02924–16. doi: 10.1128/AEM.02924-16 27986720 PMC5288824

[34] Cheon SA, Jung KW, Bahn YS, Kang HA. The unfolded protein response (UPR) pathway in *Cryptococcus*. Virulence. 2014; 5(2):341–50. doi: 10.4161/viru.26774 24504058 PMC3956512

[35] Hetz C, Papa FR. The Unfolded protein response and cell fate control. Mol Cell. 2018; 69(2):169–181. doi: 10.1016/j.molcel.2017.06.017 29107536

[36] Wang C, Zhang S, Hou R, Zhao Z, Zheng Q, Xu Q, et al. Functional analysis of the kinome of the wheat scab fungus *Fusarium graminearum*. PLoS Pathog. 2011; 7(12):e1002460. doi: 10.1371/journal.ppat.1002460 22216007 PMC3245316

[37] Yun Y, Liu Z, Yin Y, Jiang J, Chen Y, Xu JR, et al. Functional analysis of the *Fusarium graminearum* phosphatome. New Phytol. 2015; 207(1):119–134. doi: 10.1111/nph.13374 25758923

[38] Hooks KB, Griffiths-Jones S. Conserved RNA structures in the non-canonical Hac1/Xbp1 intron. RNA Biol. 2011; 8(4):552–6. doi: 10.4161/rna.8.4.15396 21593604 PMC3225973

[39] Snapp EL, Hegde RS, Francolini M, Lombardo F, Colombo S, Pedrazzini E, et al. Formation of stacked ER cisternae by low affinity protein interactions. J Cell Biol. 2003; 163(2):257–69. doi: 10.1083/jcb.200306020 14581454 PMC2173526

[40] Wright R, Basson M, D’Ari L, Rine J. Increased amounts of HMG-CoA reductase induce "karmellae": a proliferation of stacked membrane pairs surrounding the yeast nucleus. J Cell Biol. 1988; 107(1):101–14. doi: 10.1083/jcb.107.1.101 3292536 PMC2115167

[41] Vergères G, Yen TS, Aggeler J, Lausier J, Waskell L. A model system for studying membrane biogenesis. Overexpression of cytochrome b5 in yeast results in marked proliferation of the intracellular membrane. J Cell Sci. 1993; 106:249–59. doi: 10.1242/jcs.106.1.249 8270629

[42] Federovitch CM, Jones YZ, Tong AH, Boone C, Prinz WA, Hampton RY. Genetic and structural analysis of Hmg2p-induced endoplasmic reticulum remodeling in *Saccharomyces cerevisiae*. Mol Biol Cell. 2008; 19(10):4506–20. doi: 10.1091/mbc.E07-11-1188 18667535 PMC2555956

[43] Yamamoto A, Masaki R, Tashiro Y. Formation of crystalloid endoplasmic reticulum in COS cells upon overexpression of microsomal aldehyde dehydrogenase by cDNA transfection. J Cell Sci. 1996; 109:1727–38. doi: 10.1242/jcs.109.7.1727 8832395

[44] Korkhov VM, Milan-Lobo L, Zuber B, Farhan H, Schmid JA, Freissmuth M, et al. Peptide-based interactions with calnexin target misassembled membrane proteins into endoplasmic reticulum-derived multilamellar bodies. J Mol Biol. 2008; 378(2):337–52. doi: 10.1016/j.jmb.2008.02.056 18367207 PMC4493858

[45] Lingwood D, Schuck S, Ferguson C, Gerl MJ, Simons K. Generation of cubic membranes by controlled homotypic interaction of membrane proteins in the endoplasmic reticulum. J Biol Chem. 2009; 284(18):12041–8. doi: 10.1074/jbc.M900220200 19258319 PMC2673273

[46] Ferrero S, Grados-Torrez RE, Leivar P, Antolín-Llovera M, López-Iglesias C, Cortadellas N, et al. Proliferation and morphogenesis of the endoplasmic reticulum driven by the membrane domain of 3-hydroxy-3-methylglutaryl coenzyme A reductase in plant cells. Plant Physiol. 2015; 168(3):899–914. doi: 10.1104/pp.15.00597 26015445 PMC4741317

[47] Grados-Torrez RE, López-Iglesias C, Ferrer JC, Campos N. Loose Morphology and high dynamism of OSER structures induced by the membrane domain of HMG-CoA reductase. Int J Mol Sci. 2021; 22(17):9132. doi: 10.3390/ijms22179132 34502042 PMC8430881

[48] Jingami H, Brown MS, Goldstein JL, Anderson RG, Luskey KL. Partial deletion of membrane-bound domain of 3-hydroxy-3-methylglutaryl coenzyme A reductase eliminates sterol-enhanced degradation and prevents formation of crystalloid endoplasmic reticulum. J Cell Biol. 1987; 104(6):1693–704. doi: 10.1083/jcb.104.6.1693 3584246 PMC2114504

[49] Sandig G, Kärgel E, Menzel R, Vogel F, Zimmer T, Schunck WH. Regulation of endoplasmic reticulum biogenesis in response to cytochrome P450 overproduction. Drug Metab Rev. 1999; 31(2):393–410. doi: 10.1081/dmr-100101926 10335443

[50] Federovitch CM, Ron D, Hampton RY. The dynamic ER: experimental approaches and current questions. Curr Opin Cell Biol. 2005; 17(4):409–14. doi: 10.1016/j.ceb.2005.06.010 15975777

[51] Korkhov VM, Zuber B. Direct observation of molecular arrays in the organized smooth endoplasmic reticulum. BMC Cell Biol. 2009; 10:59. doi: 10.1186/1471-2121-10-59 19703297 PMC2737311

[52] Sandor A, Fricker MD, Kriechbaumer V, Sweetlove LJ. IntEResting structures: formation and applications of organized smooth endoplasmic reticulum in plant cells. Plant Physiol. 2021; 185(3):550–561. doi: 10.1104/pp.20.00719 33822222 PMC8892044

[53] Chin DJ, Luskey KL, Anderson RG, Faust JR, Goldstein JL, Brown MS. Appearance of crystalloid endoplasmic reticulum in compactin-resistant Chinese hamster cells with a 500-fold increase in 3-hydroxy-3-methylglutaryl-coenzyme A reductase. Proc Natl Acad Sci U S A. 1982; 79(4):1185–9. doi: 10.1073/pnas.79.4.1185 6951166 PMC345926

[54] Larson LL, Parrish ML, Koning AJ, Wright RL. Proliferation of the endoplasmic reticulum occurs normally in cells that lack a functional unfolded protein response. Yeast. 2002; 19(4):373–92. doi: 10.1002/yea.839 11870859

[55] Menzel R, Vogel F, Kärgel E, Schunck WH. Inducible membranes in yeast: relation to the unfolded-protein-response pathway. Yeast. 1997; 13(13):1211–29. 10.1002/(SICI)1097-0061(199710)13:13<1211::AID-YEA168>3.0.CO;2-8 9364746

[56] Schuck S, Prinz WA, Thorn KS, Voss C, Walter P. Membrane expansion alleviates endoplasmic reticulum stress independently of the unfolded protein response. J Cell Biol. 2009; 187(4):525–36. doi: 10.1083/jcb.200907074 19948500 PMC2779237

[57] Koning AJ, Larson LL, Cadera EJ, Parrish ML, Wright RL. Mutations that affect vacuole biogenesis inhibit proliferation of the endoplasmic reticulum in *Saccharomyces cerevisiae*. Genetics. 2002; 160(4):1335–52. doi: 10.1093/genetics/160.4.1335 11973291 PMC1462048

[58] Liu Z, Jian Y, Chen Y, Kistler HC, He P, Ma Z, et al. A phosphorylated transcription factor regulates sterol biosynthesis in *Fusarium graminearum*. Nat Commun. 2019; 10(1):1228. doi: 10.1038/s41467-019-09145-6 30874562 PMC6420630

[59] Simpson DR, Weston GE, Turner JA, Jennings P, Nicholson P. Differential control of head blight pathogens of wheat by fungicides and consequences for mycotoxin contamination of grain. Europ J Plant Pathol. 2001; 107:421–431. 10.1023/A:1011225817707

[60] Magan N, Hope R, Colleate A, Baxter ES. Relationship between growth and mycotoxin production by *Fusarium* species, biocides and environment. Europ J Plant Pathol. 2002; 108:685–690. 10.1023/A:1020618728175

[61] Gardiner DM, Kazan K, Manners JM. Nutrient profiling reveals potent inducers of trichothecene biosynthesis in *Fusarium graminearum*. Fungal Genet Biol. 2009; 46(8):604–13. doi: 10.1016/j.fgb.2009.04.004 19406250

[62] Yu JH, Hamari Z, Han KH, Seo JA, Reyes-Domínguez Y, Scazzocchio C. Double-joint PCR: a PCR-based molecular tool for gene manipulations in filamentous fungi. Fungal Genet Biol. 2004; 41(11):973–81. doi: 10.1016/j.fgb.2004.08.001 15465386

[63] Proctor RH, Hohn TM, McCormick SP. Reduced virulence of *Gibberella zeae* caused by disruption of a trichothecene toxin biosynthetic gene. Mol Plant Microbe Interact. 1995; 8(4):593–601. doi: 10.1094/mpmi-8-0593 8589414

[64] Hou Z, Xue C, Peng Y, Katan T, Kistler HC, Xu JR. A mitogen-activated protein kinase gene (MGV1) in *Fusarium graminearum* is required for female fertility, heterokaryon formation, and plant infection. Mol Plant Microbe Interact. 2002; 15(11):1119–27. doi: 10.1094/MPMI.2002.15.11.1119 12423017

[65] Zhou X, Li G, Xu JR. Efficient approaches for generating GFP fusion and epitope-tagging constructs in filamentous fungi. Methods Mol Biol. 2011; 722:199–212. doi: 10.1007/978-1-61779-040-9_15 21590423

[66] Wang J, Xu C, Sun Q, Xu J, Chai Y, Berg G, et al. Post-translational regulation of autophagy is involved in intra-microbiome suppression of fungal pathogens. Microbiome. 2021; 9(1):131. doi: 10.1186/s40168-021-01077-y 34092253 PMC8182927

[67] Frandsen RJ, Andersson JA, Kristensen MB, Giese H. Efficient four fragment cloning for the construction of vectors for targeted gene replacement in filamentous fungi. BMC Mol Biol. 2008; 9:70. doi: 10.1186/1471-2199-9-70 18673530 PMC2533011

