# Supplementary figures and images for: Overproduction of mycotoxin biosynthetic enzymes triggers Fusarium toxisome-shaped structure formation via endoplasmic reticulum remodeling
Source: PLoS Pathog. 2024 Jan 2;20(1):e1011913. doi: 10.1371/journal.ppat.1011913 (PMC10786393; doi:10.1371/journal.ppat.1011913)

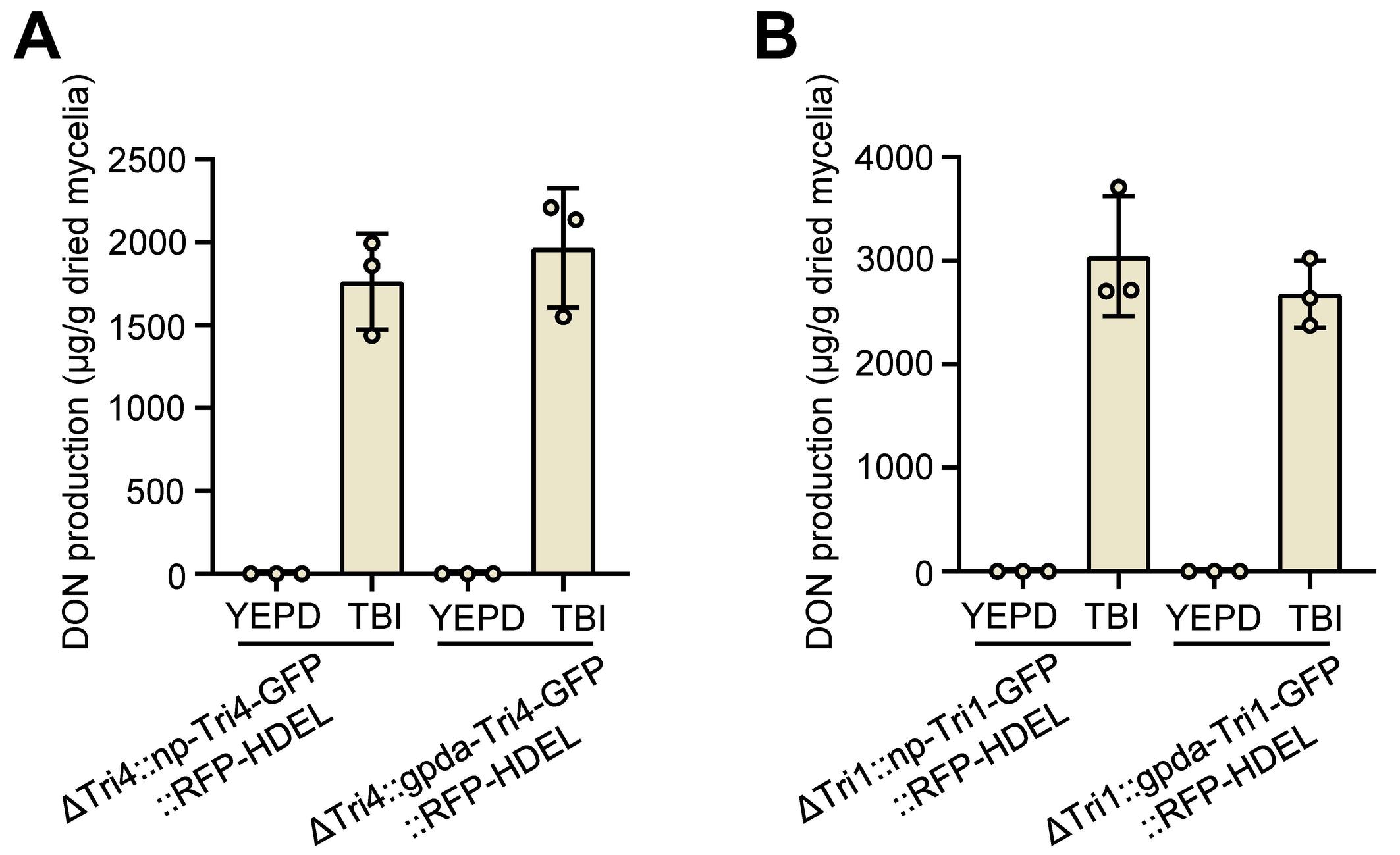

Supplement: S1 Fig — Strains ΔTri4::np-Tri4-GFP::RFP-HDEL, ΔTri4::gpda-Tri4-GFP::RFP-HDEL, ΔTri1::np-Tri1-GFP::RFP-HDEL and ΔTri1::gpda-Tri1-GFP::RFP-HDEL in Fig 1 were incubated in YEPD or TBI medium for 7 d and determined for DON production by LC-MS. Data represent the mean ± s.d. from three independent experiments. (TIF) [file ppat.1011913.s001.tif]

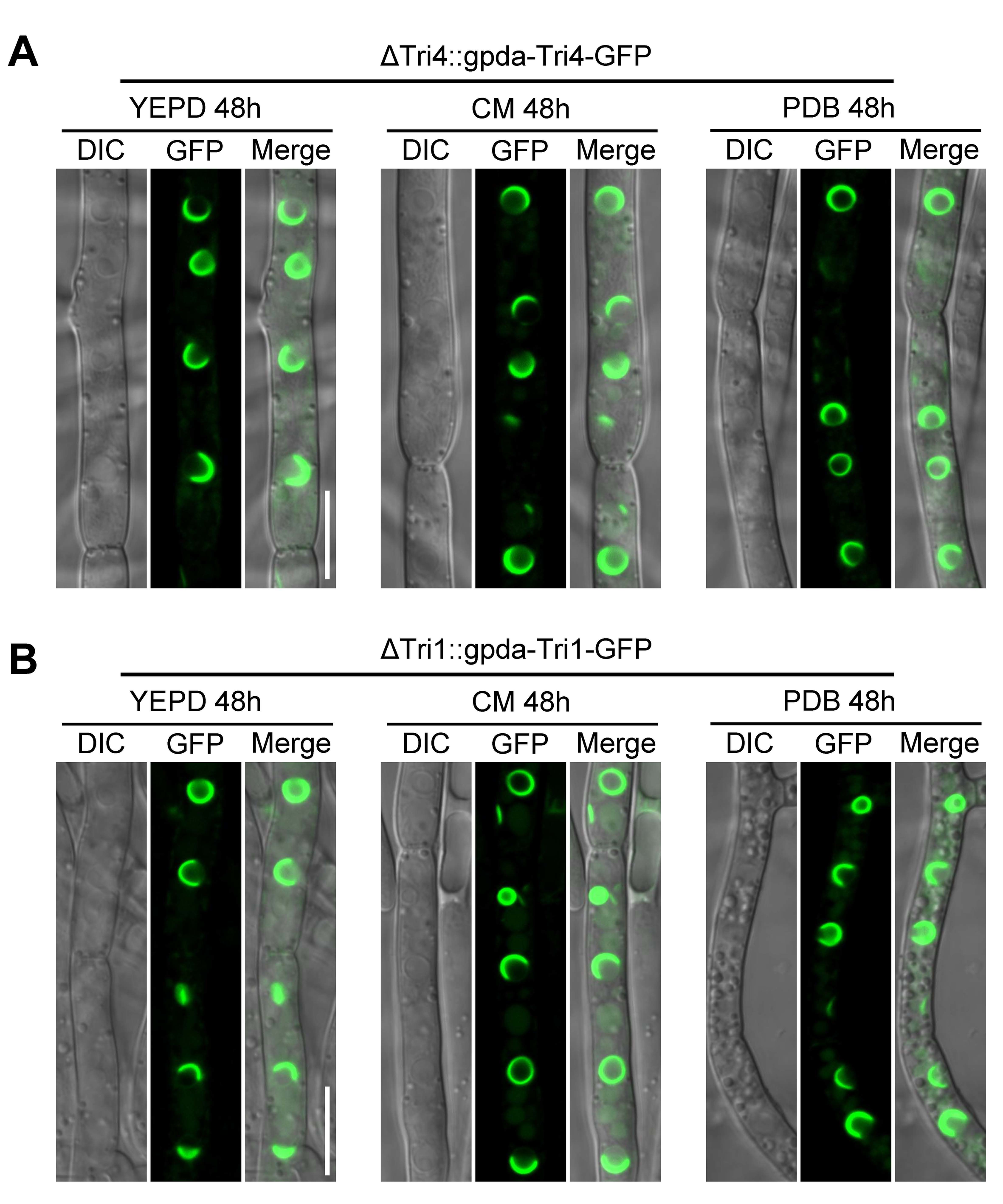

Supplement: S2 Fig — Toxisome-shaped structures were formed in the mycelia of strains ΔTri4::gpda-Tri4-GFP (A) and ΔTri1::gpda-Tri1-GFP (B) after 48 h incubation at 28°C in CM and PDB liquid media. Bar = 10 μm. (TIF) [file ppat.1011913.s002.tif]

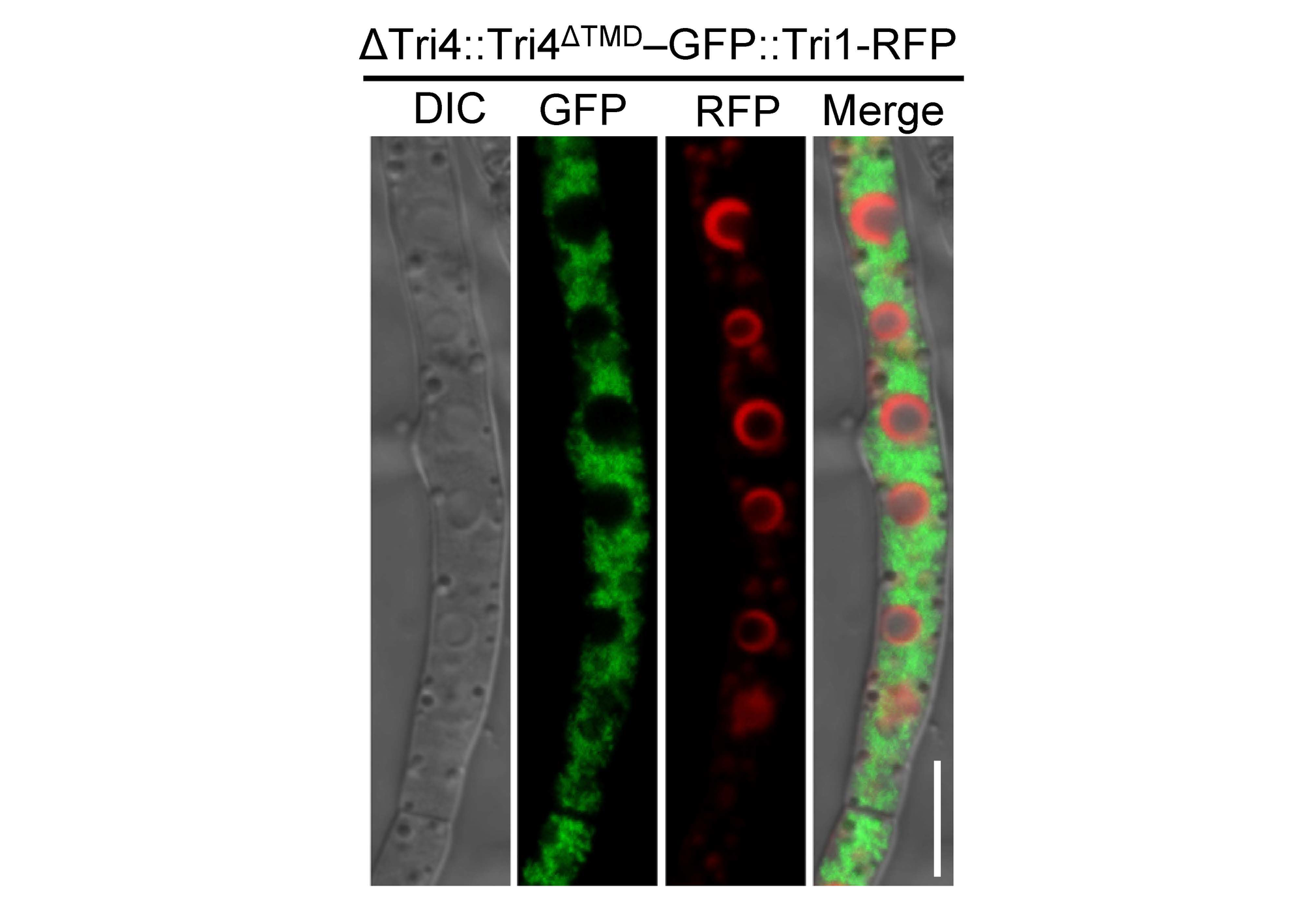

Supplement: S3 Fig — The dual-labelled strain was grown in TBI medium at 28°C for 48 h before observation. Bar = 10 μm. (TIF) [file ppat.1011913.s003.tif]

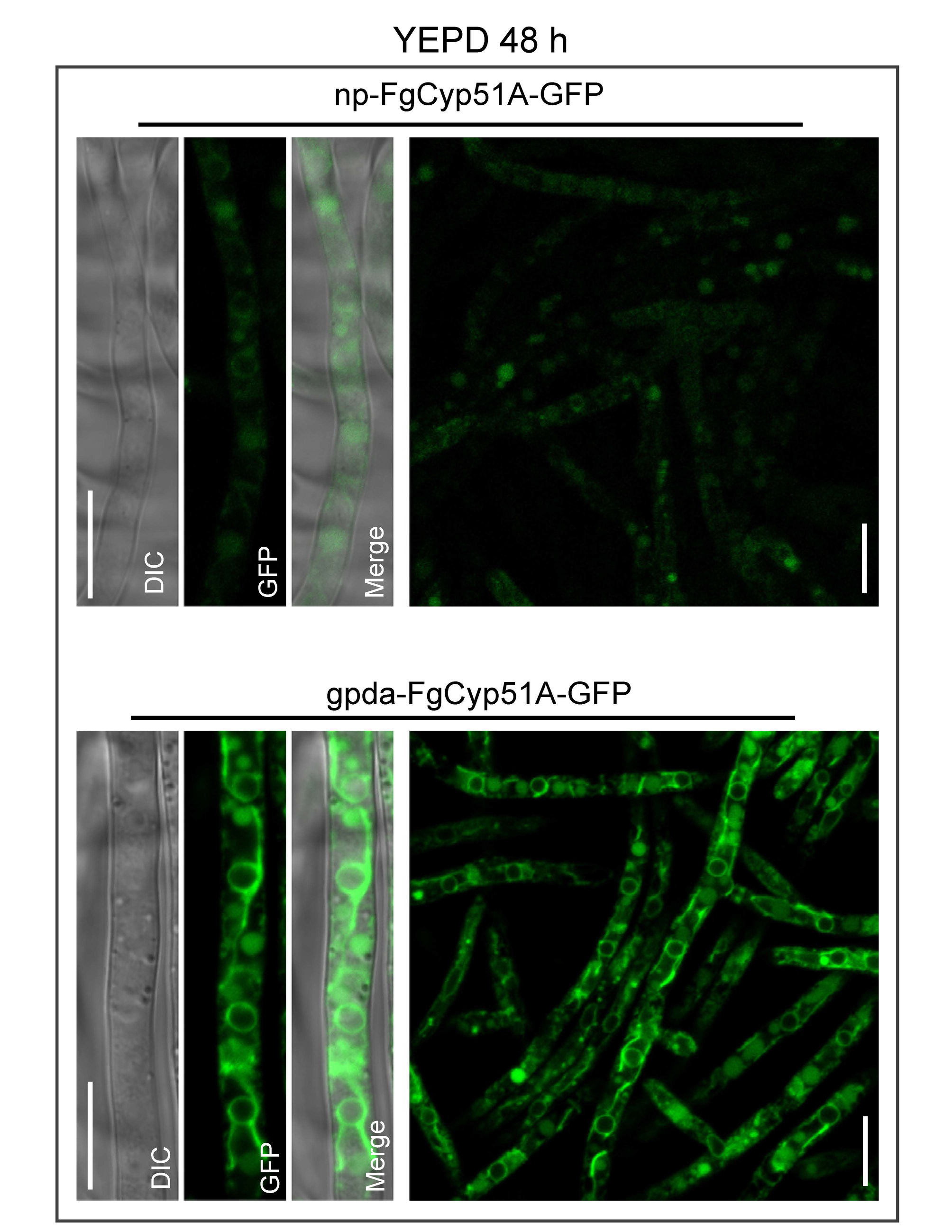

Supplement: S4 Fig — Localization of FgCyp51A-GFP under native promoter (upper panels) and gpda promoter (lower panels) in the non-inducing YEPD medium. Each strain was grown in YEPD medium at 28°C for 48 h. Bar = 10 μm. (TIF) [file ppat.1011913.s004.tif]

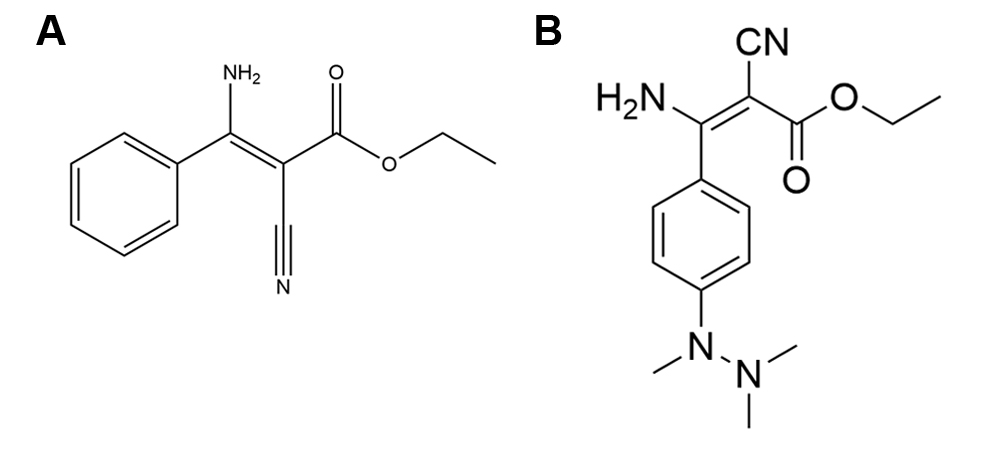

Supplement: S5 Fig — Chemical structures of the fungicide phenamacril (A) and its derivative ZJU212 (B). (TIF) [file ppat.1011913.s005.tif]

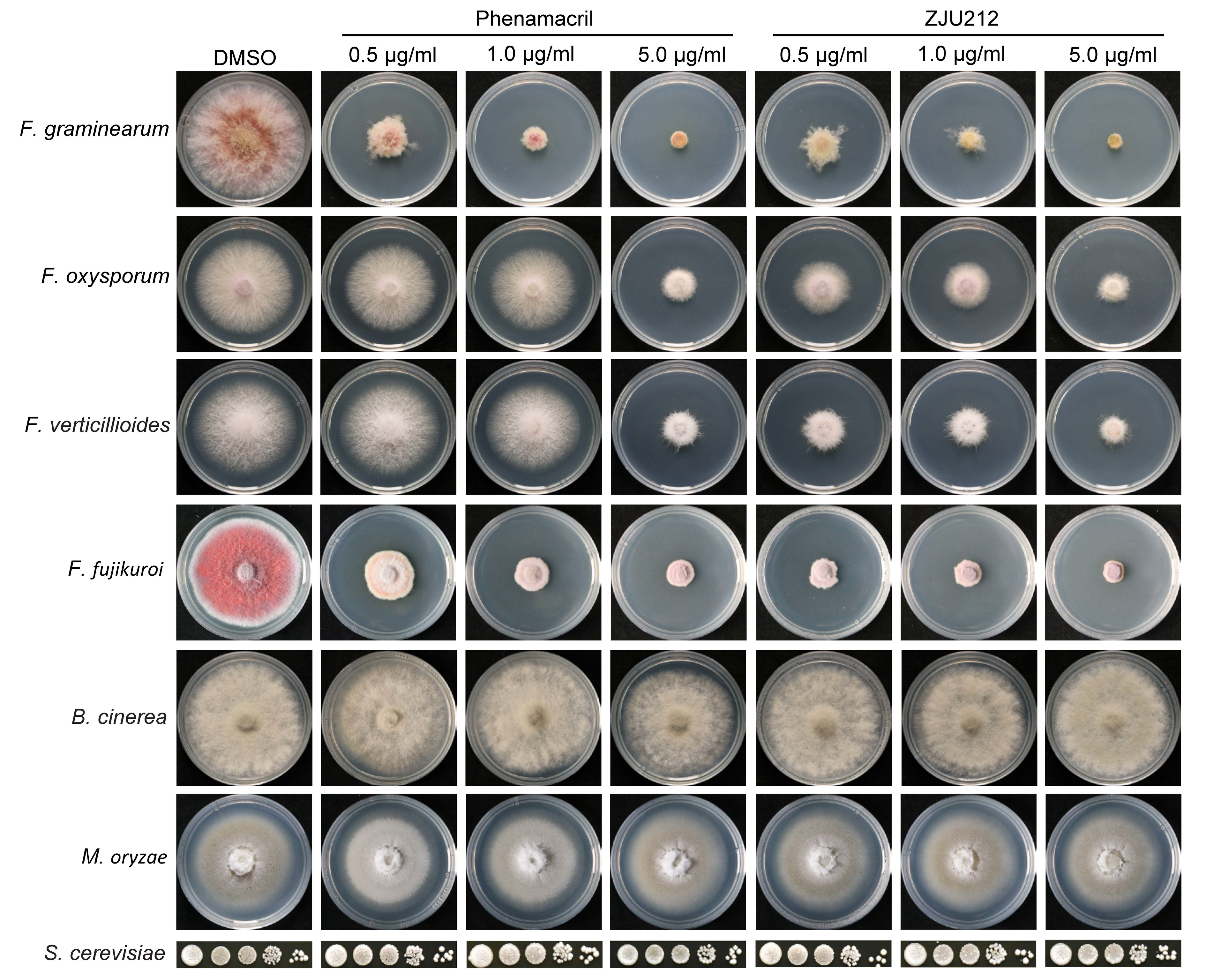

Supplement: S6 Fig — Fusarium graminearum, Fusarium oxysporum f. sp. lycopersici, Fusarium verticillioides, Fusarium fujikuroi, Botrytis cinerea, Magnaporthe oryzae, and Saccharomyces cerevisiae were inoculated on PDA plates supplemented with various concentrations of ZJU212 and phenamacril as indicated. The inoculated plates were incubated at 25°C and imaged when mycelia on the control plate extended to the edge of plates. S. cerevisiae was grown on the YPDA plate amended with different concentrations of ZJU212 and phenamacril, and incubated at 30°C for 3 days before imaging. DMSO was used as a negative control. (TIF) [file ppat.1011913.s006.tif]

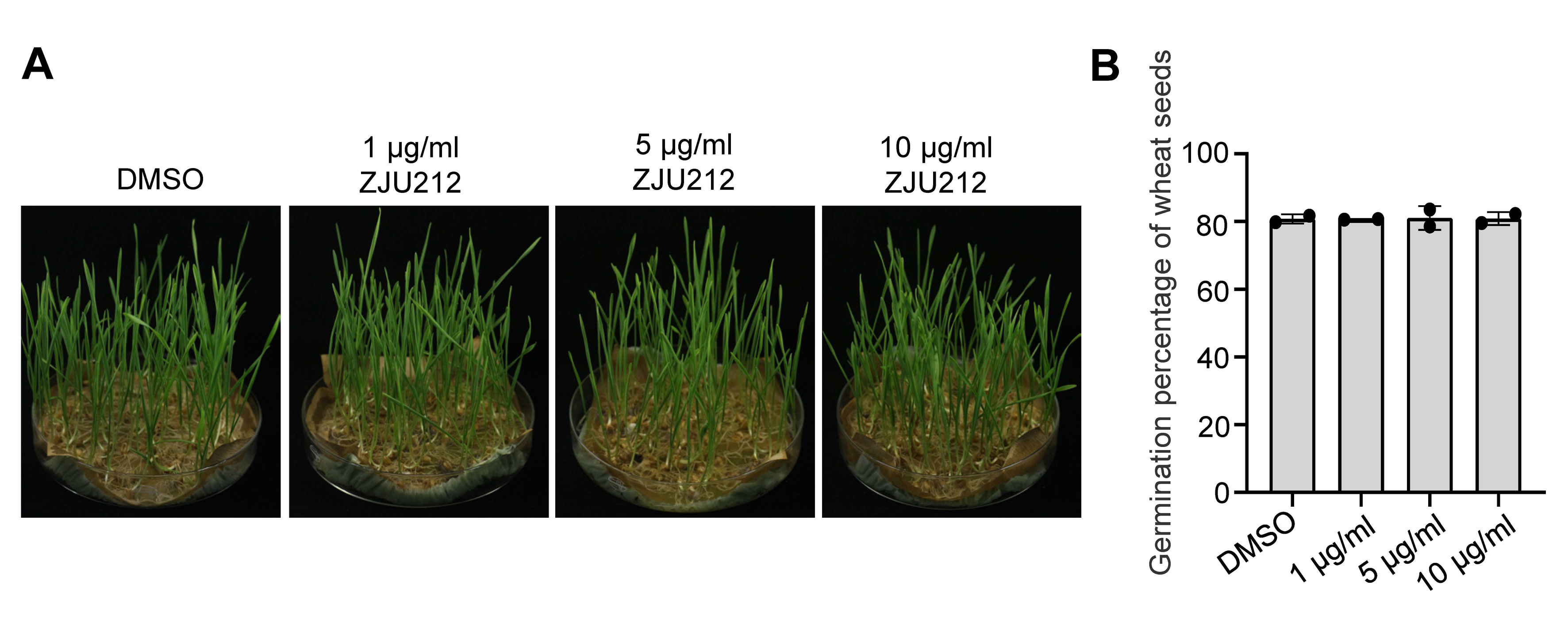

Supplement: S7 Fig — (A) The effects of ZJU212 on wheat seedings. Wheat seeds were soaked in H2O supplemented with ZJU212 at a final concentration of 1, 5, and 10 μg/mL at 25°C for 24 h, then incubated for 7 days and photographed. The solvent DMSO was used as a non-treatment control. (B) The effects of ZJU212 on wheat seed germination. Wheat seeds were soaked in H2O supplemented with ZJU212 at the indicated concentration at 25°C for 24 h, and after another 3 days incubation, the seed germination percentage of each treatment was measured. (TIF) [file ppat.1011913.s007.tif]

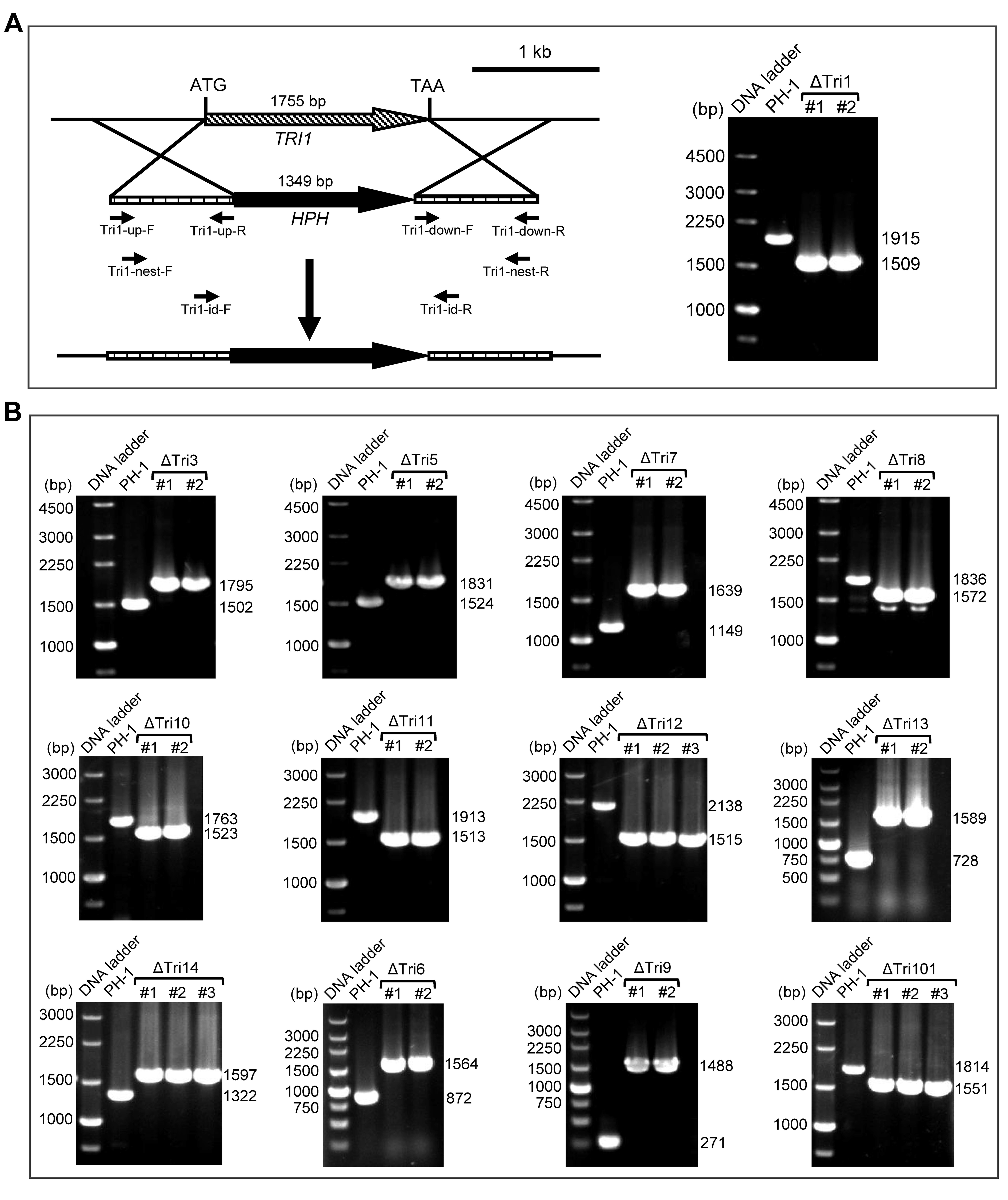

Supplement: S8 Fig — (A) Schematic representation of the TRI1 disruption strategy (left panel) and PCR assays for identification of TRI1 gene deletion mutant (right panel). Binding positions of PCR primers (arrows indicated) used for the construction of TRI1 deletion mutant are illustrated (left panel). Correct deletion was confirmed in two independent ΔTri1 transformants (#1–2) by PCR assay (right panel) using the primer pairs Tri1-id-F/Tri1-id-R indicated in left panel. Strain #2 was used for further studies. (B) Validation of TRI cluster gene deletion mutants in the parental strain PH-1::np-Tri4-GFP::RFP-HDEL by PCR assays. Correct deletion was confirmed in two or three independent deletion transformants by PCR assay using the primer pairs listed in S1 Table. (TIF) [file ppat.1011913.s008.tif]

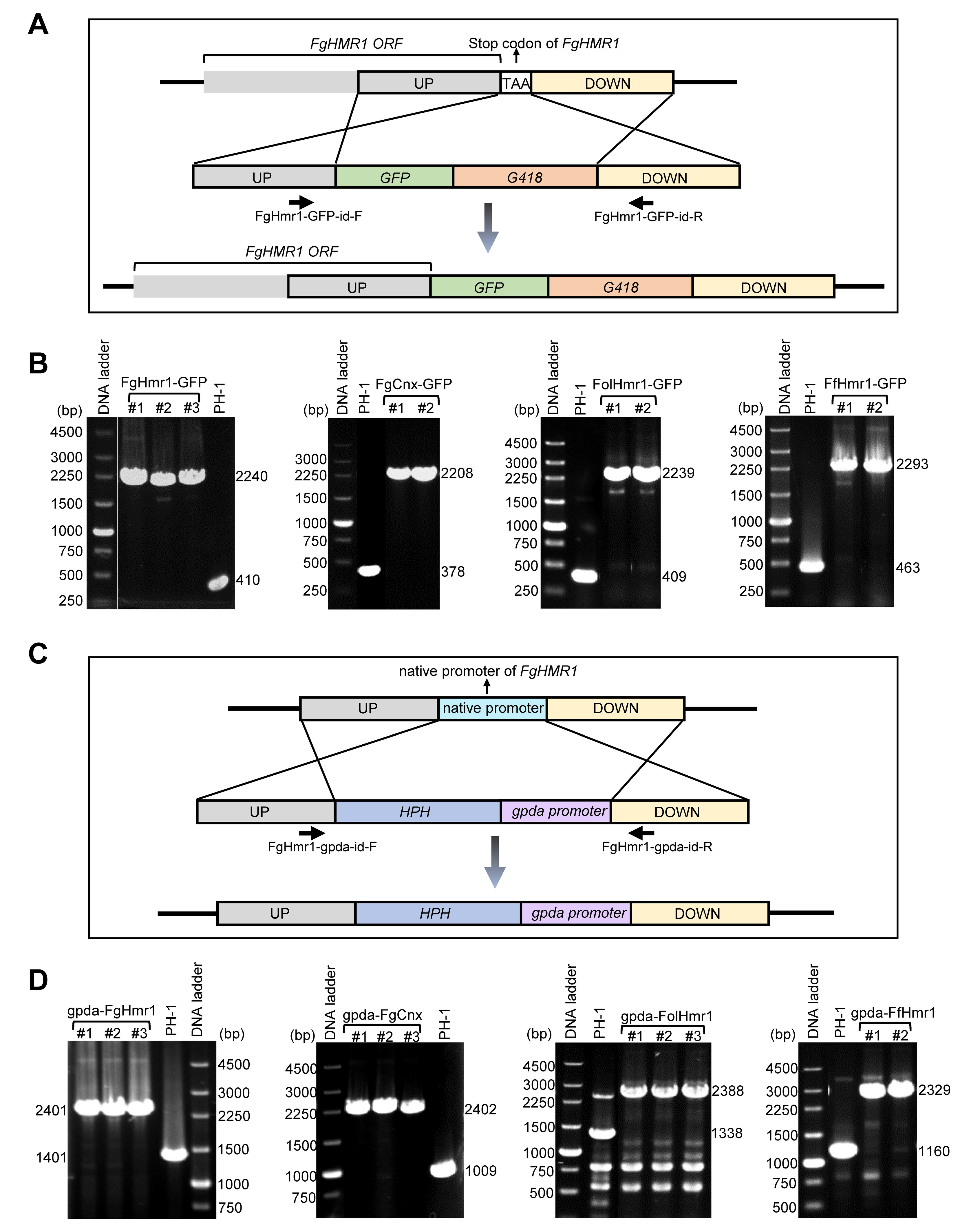

Supplement: S9 Fig — (A) Schematic representation of the knock-in strategy to generate the strain np-FgHmr1-GFP (expressing FgHmr1-GFP under the native promoter). By homologous recombination between flanking regions upstream (UP) and downstream (DOWN) of the stop codon of FgHMR1 and homologous flanks of the UP-GFP-G418-DOWN construct, the stop codon is replaced by the GFP-G418 fusion fragment of the construct. The strains np-FgCnx-GFP, np-FolHmr1-GFP and np-FfHmr1-GFP driven by native promoters were constructed using the same strategy. (B) Identification of the correct transformants that expess FgHmr1-GFP under native promoter by using PCR primers as indicated in (A) (arrows). The strains np-FgCnx-GFP, np-FolHmr1-GFP and np-FfHmr1-GFP were also identified with primers listed in S1 Table. (C) Schematic representation of the strategy to generate the strain gpda-FgHmr1-GFP (expressing FgHmr1-GFP under the gpda promoter). By homologous recombination between the 5’ and 3’ flanking regions (UP and DOWN) of the native promoter of FgHmr1 in the strain np-FgHmr1-GFP and homologous flanks of the UP-HPH-gpda-DOWN construct, the native promoter is replaced by the HPH-gpda fusion fragment. The strains gpda-FgCnx-GFP, gpda-FolHmr1-GFP and gpda-FfHmr1-GFP driven by gpda promoters were constructed using the same strategy. (D) Identification of the correct transformants that expessing FgHmr1-GFP under gpda promoter by using PCR primers as indicated in (C) (arrows). The strains gpda-FgCnx-GFP, gpda-FolHmr1-GFP and gpda-FfHmr1-GFP were also identified with primers listed in S1 Table. (TIF) [file ppat.1011913.s009.tif]
