# Supplementary material for: Overproduction of mycotoxin biosynthetic enzymes triggers Fusarium toxisome-shaped structure formation via endoplasmic reticulum remodeling
Source: PLoS Pathog. 2024 Jan 2;20(1):e1011913. doi: 10.1371/journal.ppat.1011913 (PMC10786393; doi:10.1371/journal.ppat.1011913)
Supplement: S1 Table — (DOC) [file ppat.1011913.s010.doc]

**Table S1**. **PCR primers used in this study**

| **Primer** | **Sequence (5’-3’)** | **Relevant characteristics** |
| --- | --- | --- |
| Tri1-UP-F  Tri1-UP-R | CCTGAGAGTCAGGCCTACC  CAAAATAGGCATTGATGTGTTGACCTCCGACAGCGAAATGGTCTGTCAAG | PCR primers to amplify *TRI1* upstream fragment for the construction of *TRI1*deletion mutants |
| Tri1-DOWN-F  Tri1-DOWN-R | CTCGTCCGAGGGCAAAGGAATAGAGTAGGTAGGAGGACGTCACAGTCTT  CGCTGGTAGTCTCAGTCATG | PCR primers to amplify *TRI1* downstream fragment for the construction of *TRI1* deletion mutants |
| Tri1-Nest-F  Tri1-Nest-R | CCTATAGAGAGGTTGGAGGC  CAGAAATACAGATACTTACGCGC | PCR primers to amplify *TRI1* deletion fragment for the construction of *TRI1* deletion mutants |
| Tri1-ID-F  Tri1-ID-R | TAGGATACCCTCTACAACTCTG  GTCCTCCACTCGAGGCAAA | PCR primers for identification of *TRI1* deletion transformants |
| Tri3-UP-F  Tri3-UP-R | GCGGCTATACGGACCATGTC  CAAAATAGGCATTGATGTGTTGACCTCCGATGGCAAGGTTGTACTGGTAAC | PCR primers to amplify *TRI3* upstream fragment for the construction of *TRI3* deletion mutants |
| Tri3-DOWN-F  Tri3-DOWN-R | CTCGTCCGAGGGCAAAGGAATAGAGTAGCCAATGTCGGCGCCGTTG  CAACGACGGTACCTTTCAGCC | PCR primers to amplify *TRI3* downstream fragment for the construction of *TRI3* deletion mutants |
| Tri3-Nest-F  Tri3-Nest-R | CGGGATTCGGGATTCTGTAC  GCAGCTTCTGAGACTCCTGG | PCR primers to amplify *TRI3* deletion fragment for the construction of *TRI3* deletion mutants |
| Tri3-ID-F  Tri3-ID-R | CTTGACTAACAACTGCATACGGG  CTCAGGTTACGTACAGACATTC | PCR primers for identification of *TRI3* deletion transformants |
| Tri5-UP-F  Tri5-UP-R | GTCAAGACGGCAGACAGCG  CAAAATAGGCATTGATGTGTTGACCTCCGATGGCAAGGTTGTACTGGTAAC | PCR primers to amplify *TRI5* upstream fragment for the construction of *TRI5* deletion mutants |
| Tri5-DOWN-F  Tri5-DOWN-R | CTCGTCCGAGGGCAAAGGAATAGAGTAGCTTTGAGCAGGCGGCCAATG  GTACCTTTCAGCCTTGTGATGC | PCR primers to amplify *TRI5* downstream fragment for the construction of *TRI5* deletion mutants |
| Tri5-Nest-F  Tri5-Nest-R | GGGATTCTGTACTCTGTACTCTG  CTGAGACTCCTGGAGTGCAAG | PCR primers to amplify *TRI5* deletion fragment for the construction of *TRI5* deletion mutants |
| Tri5-ID-F  Tri5-ID-R | GACTTTGGATCAGTCTTTAGGCC  CAGACATTCAGTTTCAACTGCC | PCR primers for identification of *TRI5* deletion transformants |
| Tri6-UP-F  Tri6-UP-R | CCGTGTCAGATAAGCTTCG  CAAAATAGGCATTGATGTGTTGACCTCCTTCGAGGGTAGTCAAAATAGATG | PCR primers to amplify *TRI6* upstream fragment for the construction of *TRI6* deletion mutants |
| Tri6-DOWN-F  Tri6-DOWN-R | CTCGTCCGAGGGCAAAGGAATAGAGTAGCGAGGGATCGGTGTGCAAAAC  AACAGTGTTTGACTCAGTGC | PCR primers to amplify *TRI6* downstream fragment for the construction of *TRI6* deletion mutants |
| Tri6-Nest-F  Tri6-Nest-R | AGGATGAACAAGGGGTCTG  GACCCCAAAGACACAATCCA | PCR primers to amplify *TRI6* deletion fragment for the construction of *TRI6* deletion mutants |
| Tri6-ID-F  Tri6-ID-R | TCCCATCAAGGCTCAAGCC  GCGCCAAACTCGTCATCAT | PCR primers for identification of *TRI6* deletion transformants |
| Tri7-UP-F  Tri7-UP-R | GAGGAAGGGCTAAGGACGTAT  CAAAATAGGCATTGATGTGTTGACCTCCGGTGGTCGTGTAAACGATATC | PCR primers to amplify *TRI7* upstream fragment for the construction of *TRI7* deletion mutants |
| Tri7-DOWN-F  Tri7-DOWN-R | CTCGTCCGAGGGCAAAGGAATAGAGTAGGGTCTAAATACCTTTTAAGGGAG  CCAGTCCAACAATTGCCTCAG | PCR primers to amplify *TRI7* downstream fragment for the construction of *TRI7* deletion mutants |
| Tri7-Nest-F  Tri7-Nest-R | GTCCTGGGTTCTTTAAGTGTAG  GCTGGTACCAAGGGTGGCAA | PCR primers to amplify *TRI7* deletion fragment for the construction of *TRI7* deletion mutants |
| Tri7-ID-F  Tri7-ID-R | AGGATCTTTGTGACCACCCC  GCTTACTCCATCAGATCGCCG | PCR primers for identification of *TRI7* deletion transformants |
| Tri8-UP-F  Tri8-UP-R | CCCTCGCGAGTTGAATAGGAC  CAAAATAGGCATTGATGTGTTGACCTCCGAGGAAGGGCTAAGGACG | PCR primers to amplify *TRI8* upstream fragment for the construction of *TRI8* deletion mutants |
| Tri8-DOWN-F  Tri8-DOWN-R | CTCGTCCGAGGGCAAAGGAATAGAGTAGTTTCATTTCTTGACATCTCTGGCC  CATCGCTCCATATAGCTAACCG | PCR primers to amplify *TRI8* downstream fragment for the construction of *TRI8* deletion mutants |
| Tri8-Nest-F  Tri8-Nest-R | CAACGTTTCGAGGCCCAAG  GGCATAAGGTAAGGTGCCTC | PCR primers to amplify *TRI8* deletion fragment for the construction of *TRI8* deletion mutants |
| Tri8-ID-F  Tri8-ID-R | GGACATGGGGACAATAAATCGG  GGCGTACCAAGTCGATGACC | PCR primers for identification of *TRI8* deletion transformants |
| Tri9-UP-F  Tri9-UP-R | CTGTCTCGCGTCGCTGTG  CAAAATAGGCATTGATGTGTTGACCTCCCTTGGAGATTGGGGGTGAGTG | PCR primers to amplify *TRI9* upstream fragment for the construction of *TRI9* deletion mutants |
| Tri9-DOWN-F  Tri9-DOWN-R | CTCGTCCGAGGGCAAAGGAATAGAGTAGGACTGCAGTGCCAGATCAGC  CTACCGACAGACGAGACGC | PCR primers to amplify *TRI9* downstream fragment for the construction of *TRI9* deletion mutants |
| Tri9-Nest-F  Tri9-Nest-R | GTGGATAGGTCTGAAGAATCGG  CTCATCACTCAGATTTACGCCG | PCR primers to amplify *TRI9* deletion fragment for the construction of *TRI9* deletion mutants |
| Tri9-ID-F  Tri9-ID-R | GAACACGACATCAATTGCATCAC  GGCAATTCTTCCTCTGTTGGG | PCR primers for identification of *TRI9* deletion transformants |
| Tri10-UP-F  Tri10-UP-R | CTCTATGGCCCAAGGACCTG  CAAAATAGGCATTGATGTGTTGACCTCCGCAAGGGTATGGTCACTGATC | PCR primers to amplify *TRI10* upstream fragment for the construction of *TRI10* deletion mutants |
| Tri10-DOWN-F  Tri10-DOWN-R | CTCGTCCGAGGGCAAAGGAATAGAGTAGGATGGTATCGGCGCAGAGAC  GACGTTTCAGCTTCACACCAAC | PCR primers to amplify *TRI10* downstream fragment for the construction of *TRI10* deletion mutants |
| Tri10-Nest-F  Tri10-Nest-R | GGGTCAATGATCTCATGTCATTC  CCATCGGCCAGAACCGATTAG | PCR primers to amplify *TRI10* deletion fragment for the construction of *TRI10* deletion mutants |
| Tri10-ID-F  Tri10-ID-R | GAGGTTGGAGGGAGCTGG  GCATCAAACTTACATGTATCGGG | PCR primers for identification of *TRI10* deletion transformants |
| Tri11-UP-F  Tri11-UP-R | GCCATCTGACTTCAGCCCTG  CAAAATAGGCATTGATGTGTTGACCTCCGACGTCTGAATATTCGAAGAGTGC | PCR primers to amplify *TRI11* upstream fragment for the construction of *TRI11* deletion mutants |
| Tri11-DOWN-F  Tri11-DOWN-R | CTCGTCCGAGGGCAAAGGAATAGAGTAGGGCTGCCGGCCTCAAAGC  GAATATGCAAGCGTCGAACCG | PCR primers to amplify *TRI11* downstream fragment for the construction of *TRI11* deletion mutants |
| Tri11-Nest-F  Tri11-Nest-R | CCCAGTCTTCTTGAGGCTGC  GAATGCCATCGAGCTTCACG | PCR primers to amplify *TRI11* deletion fragment for the construction of *TRI11* deletion mutants |
| Tri11-ID-F  Tri11-ID-R | CGTTGAACCTAAGACGTGAACC  GACAGATCAGCAAACATGGAGC | PCR primers for identification of *TRI11* deletion transformants |
| Tri12-UP-F  Tri12-UP-R | GGGCACGCGTGGTTGTTTTG  CAAAATAGGCATTGATGTGTTGACCTCCCTTTGAACGAATAGGGCGGTC | PCR primers to amplify *TRI12* upstream fragment for the construction of *TRI12* deletion mutants |
| Tri12-DOWN-F  Tri12-DOWN-R | CTCGTCCGAGGGCAAAGGAATAGAGTAGCATAACTGTCCGGCATGCAGC CAGTACTATGATTGGGTCCTTG | PCR primers to amplify *TRI12* downstream fragment for the construction of *TRI12* deletion mutants |
| Tri12-Nest-F  Tri12-Nest-R | CACCATCGAAGTTCTGACTAGC  GCATGTTTGGCCCAATACGG | PCR primers to amplify *TRI12* deletion fragment for the construction of *TRI12* deletion mutants |
| Tri12-ID-F  Tri12-ID-R | CCATCGGGAGTGCTAGATAC  GTTGCTGACTTGCCAGTCTCG | PCR primers for identification of *TRI12* deletion transformants |
| Tri13-UP-F  Tri13-UP-R | GCCCCAGTCTCACATAGAAG  CAAAATAGGCATTGATGTGTTGACCTCCCGTTGACAATTAATTTCGGCTCC | PCR primers to amplify *TRI13* upstream fragment for the construction of *TRI13* deletion mutants |
| Tri13-DOWN-F  Tri13-DOWN-R | CTCGTCCGAGGGCAAAGGAATAGAGTAGTTGAACTTTGAGCTGCTGCC  GAACGAATAGGGCGGTCGATA | PCR primers to amplify *TRI13* downstream fragment for the construction of *TRI13* deletion mutants |
| Tri13-Nest-F  Tri13-Nest-R | TCTTCCCAAGCCAATACCGAG  GCACTCCCGATGGTCGTAAG | PCR primers to amplify *TRI13* deletion fragment for the construction of *TRI13* deletion mutants |
| Tri13-ID-F  Tri13-ID-R | CTGCCACTTCTGCGTTGGAAC  GAAGTCTACTCTGCCCTTGTC | PCR primers for identification of *TRI13* deletion transformants |
| Tri14-UP-F  Tri14-UP-R | GATTACCCAGTGGGTTGAGAAAC  CAAAATAGGCATTGATGTGTTGACCTCCGGTGGGCATTGTGTGCTG | PCR primers to amplify *TRI14* upstream fragment for the construction of *TRI14* deletion mutants |
| Tri14-DOWN-F  Tri14-DOWN-R | CTCGTCCGAGGGCAAAGGAATAGAGTAGGGCTCAACTGTAAGATGATGGAG  GGTCACCCTGATCTTGCAGC | PCR primers to amplify *TRI14* downstream fragment for the construction of *TRI14* deletion mutants |
| Tri14-Nest-F  Tri14-Nest-R | GCCGCCTTGATCGTAGCTG  GTAAGGACCCTGTCTACATGC | PCR primers to amplify *TRI14* deletion fragment for the construction of *TRI14* deletion mutants |
| Tri14-ID-F  Tri14-ID-R | CTCTGCCCTCTACATATCTATC  GTCTGTGTATGTCGGAGTGAC | PCR primers for identification of *TRI14* deletion transformants |
| Tri101-UP-F  Tri101-UP-R | GGAGTAAGTCAATTACGACCGG  CAAAATAGGCATTGATGTGTTGACCTCCGCTTCTTTAGGCGACTCAGG | PCR primers to amplify *TRI101* upstream fragment for the construction of *TRI101* deletion mutants |
| Tri101-DOWN-F  Tri101-DOWN-R | CTCGTCCGAGGGCAAAGGAATAGAGTAGCAAACACTTTCCACTTTGCTGTAC  CATCATCGTCCTTGCCCTGTC | PCR primers to amplify *TRI101* downstream fragment for the construction of *TRI101* deletion mutants |
| Tri101-Nest-F  Tri101-Nest-R | CTCCGAGGCTTGTACGGAG  GTCTTGGAGGGTGTCTACTTG | PCR primers to amplify *TRI101* deletion fragment for the construction of *TRI101* deletion mutants |
| Tri101-ID-F  Tri101-ID-R | GGCAGTTTGTGTTTGAAGCCATC  CGCAGGTTCCTGGCTAAAAC | PCR primers for identification of *TRI101* deletion transformants |
| HPH-F  HPH-R | GGAGGTCAACACATCAATGCCTATT  CTACTCTATTCCTTTGCCCT | PCR primers for amplification of hygromycin resistance gene (*HPH*) |
| np-Tri1-GFP-F  np-Tri1-GFP-R | ACTCACTATAGGGCGAATTGGGTACTCAAATTGGTTCTCGATAAACCCGAGTAGACA  CACCACCCCGGTGAACAGCTCCTCGCCCTTGCTCACGTCATCCTGTACCAATTCCAATC | PCR primers to amplify the native promoter and the ORF of *TRI1* for Tri1-GFP fusion protein construction under native promoter |
| np-Tri4-GFP-F  np-Tri4-GFP-R | ACTCACTATAGGGCGAATTGGGTACTCAAATTGGTTTAACAGAGCGCTTCCGAACGG  CACCACCCCGGTGAACAGCTCCTCGCCCTTGCTCACCAAAGCCTTGAGAACCTTGACTC | PCR primers to amplify the native promoter and the ORF of *TRI4* for Tri4-GFP fusion protein construction under native promoter |
| Gpda-F  Gpda-R | TGCGGAGAGACGGACGGACG  GGTGATGTCTGCTCAAGCGG | PCR primers for amplification of the gpda promoter from *Aspergillus nidulans* |
| gpda-Tri4-GFP-fusionF  gpda-Tri4-GFP-fusionR | CTACCCCGCTTGAGCAGACATCACCATGATTGACCAAGATTGGATCAAG  CAAAGCCTTGAGAACCTTGAC | PCR primers for amplification of the fragment of *TRI4* ORF that could fused to the gpda promoter |
| gpda-Tri4-GFP-nestF  gpda-Tri4-GFP-nestR | ACTCACTATAGGGCGAATTGGGTACTCAAATTGGTTTGCGGAGAGACGGACGGACG CACCACCCCGGTGAACAGCTCCTCGCCCTTGCTCACCAAAGCCTTGAGAACCTTGACTC | PCR primers to amplify the gpda-Tri4 fusion fragment for Tri4-GFP fusion protein construction under gpda promoter |
| gpda-Tri1-GFP-fusionF  gpda-Tri1-GFP-fusionR | CTACCCCGCTTGAGCAGACATCACCATGGCTCTCATCACCAGTTTGC  GTCATCCTGTACCAATTCCAATC | PCR primers for amplification of the fragment of *TRI1* ORF that could be fused to the gpda promoter |
| gpda-Tri1-GFP-F  gpda-Tri1-GFP-R | ACTCACTATAGGGCGAATTGGGTACTCAAATTGGTTTGCGGAGAGACGGACGGACG  CACCACCCCGGTGAACAGCTCCTCGCCCTTGCTCACGTCATCCTGTACCAATTCCAATC | PCR primers to amplify the gpda-Tri1 fusion fragment for Tri1-GFP fusion protein construction under gpda promoter |
| np-Tri4^∆TM^-GFP-F1  np-Tri4^∆TM^-GFP-R1 | ACTCACTATAGGGCGAATTGGGTACTCAAATTGGTTTAACAGAGCGCTTCCGAACGG  GTTGAGCAAGCTCTTGATCC | PCR primers to amplify the fragment 1 (native promoter of *TRI4* and the 1-12 aa of Tri4 protein) for TRI4^∆TM^-GFP fusion protein construction under native promoter |
| np-Tri4^∆TM^-GFP-F2  np-Tri4^∆TM^-GFP-R2 | AGATTGGATCAAGAGCTTGCTCAACTACAACCTGTACTTGCACCCAC  CACCACCCCGGTGAACAGCTCCTCGCCCTTGCTCACCAAAGCCTTGAGAACCTTGACTC | PCR primers to amplify the fragment 2 (the 36-520 aa of Tri4 protein) for TRI4^∆TM^-GFP fusion protein construction under native promoter |
| np-Tri4^∆TM^-GFP-F3  np-Tri4^∆TM^-GFP-R3 | ACTCACTATAGGGCGAATTG  CACCACCCCGGTGAACAGCT | PCR primers to amplify the fusion fragment of 1 and 2 for TRI4^∆TM^-GFP fusion protein construction under native promoter |
| np-Tri4^∆p450^-GFP-F  np-Tri4^∆p450^-GFP-R | ACTCACTATAGGGCGAATTGGGTACTCAAATTGGTTTAACAGAGCGCTTCCGAACGG  CACCACCCCGGTGAACAGCTCCTCGCCCTTGCTCACGATCTTTCTCAGTGGGTGCAA | PCR primers to amplify the open reading frame of TRI4^∆p450^ for GFP fusion protein construction under native promoter |
| RP27-F  RP27-R | ATAAATGTAGGTATTACCTGTACATT  TTTGAAGATTGGGTTCCTACG | PCR primers for amplification of the RP27 promoter from the plasmid pYF11 |
| Bip-SP-F  Bip-SP-R | CTTTCGTAGGAACCCAATCTTCAAAATGGCTCGTTCAAGGAGCTC  GGCCTGGACAGTCTGAACGA | PCR primers for amplification of the ER targeting signal peptide sequence from *F. graminearum* BiP protein (gene locus FGSG_09471, 1-33 aa in N-terminus) with the adaptor that could be fused to the RP27 promoter |
| RFP-HDEL-F  RFP-HDEL-R | GGCTTTCGTTCAGACTGTCCAGGCCGCCTCCTCCGAGGACGTCAT  TTACAACTCGTCGTGGGCGCCGGTGGAGTGGCGG | PCR primers for amplification of the RFP-HDEL fragment with the adaptor that could be fused to the signal peptide sequence of Bip |
| Nat-fusionRFP-F  Nat-fusionRFP-R | CTCCACCGGCGCCCACGACGAGTTGTAAACTAGTGATATTGAAGGAGC  AGGCCTGATGCTTTGGTTTAG | PCR primers for amplification of the nourseothricin resistance gene fragment with the adaptor that could be fused to the RFP-HDEL fragment |
| RP27-BiP-RFP-HDEL-NAT1-nestF  RP27-BiP-RFP-HDEL-NAT1-nestR | ATAAATGTAGGTATTACCTGTACATT  AGGCCTGATGCTTTGGTTTAG | PCR primers for amplification of the RP27-BiP-RFP-HDEL-NAT1 fusion fragment |
| FgHmr1-GFP-UP-F  FgHmr1-GFP-UP-R | CAACTCGACCAGTCGATTCG  TGAACAGCTCCTCGCCCTTGCTCACTCGCTTTGACCTCTGGATGG | PCR primers to amplify upstream fragment flanking the stop codon of *FgHMR1* for the construction of FgHmr1-GFP expression strain under native promoter |
| FgHmr1-GFP-DOWN-F  FgHmr1-GFP-DOWN-R | CTATCGCCTTCTTGACGAGTTCTTCTGAATGATGAGCGTTTGCTTTTG  AGAGCGACAAGACCACTACT | PCR primers to amplify downstream fragment flanking the stop codon of *FgHMR1* for the construction of FgHmr1-GFP expression strain under native promoter |
| FgHmr1-GFP-NEST-F  FgHmr1-GFP-NEST-R | TACTACTGGTGACGCTATGGG  CAACTCTCTACACCCACGATAAG | PCR primers to amplify the up-GFP-G418-down fusion fragment for the construction of FgHmr1-GFP expression strain under native promoter |
| FgHmr1-GFP-ID-F  FgHmr1-GFP-ID-R | CTCTGTCCTTGCTGGAGAAC  AACTCATGCTACCATATCTGC | PCR primers for identification of correct np-FgHmr1-GFP transformants |
| GFP-F  GFP-R | GTGAGCAAGGGCGAGGAGCTG  TTACTTGTACAGCTCGTCCA | PCR primers for amplification of the GFP fragment from the plasmid pYF11 |
| G418-F  G418-R | CGGCATGGACGAGCTGTACAAGTAAGGAGGTCAACACATCAATGCT  TCAGAAGAACTCGTCAAGAAG | PCR primers for amplification of the G418 fragment from the plasmid pYF11 that could be fused to the GFP fragment |
| FgHmr1-gpda-up-F  FgHmr1-gpda-up-R | CCGTCAAGATGGAGATCGAC  CAAAATAGGCATTGATGTGTTGACCTCCCTAGGTGCAATTTGCAGCAC | PCR primers to amplify upstream fragment flanking the native promoter of *FgHMR1* for the construction of FgHmr1-GFP expression strain under gpda promoter |
| FgHmr1-gpda-down-F  FgHmr1-gpda-down-R | CTACCCCGCTTGAGCAGACATCACCATGGCTTCGATACTTCTGCCG  CTTGGTGGTGACGATGAGACC | PCR primers to amplify downstream fragment flanking the native promoter of *FgHMR1* for the construction of FgHmr1-GFP expression strain under gpda promoter |
| FgHmr1-gpda-nest-F  FgHmr1-gpda-nest-R | GGCCTGAAAAGTTGACAGC  CGAGCCAGAACTTGGATCCC | PCR primers to amplify the up-HPH-gpda-down fusion fragment for the construction of FgHmr1-GFP expression strain under gpda promoter |
| FgHmr1-gpda-id-F  FgHmr1-gpda-id-R | CGATACGCCACATGCCACAT  GAGCCGCTTGGAAGCCCAT | PCR primers for identification of correct gpda-FgHmr1-GFP transformants |
| FgCnx-GFP-UP-F  FgCnx-GFP-UP-R | ATGATAAGAAGCCCGAGGAC  TGAACAGCTCCTCGCCCTTGCTCACCGACTGGCTGCGGGTGGT | PCR primers to amplify upstream fragment flanking the stop codon of *FgCNX* for the construction of FgCnx-GFP expression strain under native promoter |
| FgCnx-GFP-DOWN-F  FgCnx-GFP- DOWN-R | CTATCGCCTTCTTGACGAGTTCTTCTGAGGAGAAGACGGAAACTGGGC  TGAGAAATTGGCCTACCTGCA | PCR primers to amplify downstream fragment flanking the stop codon of *FgCNX* for the construction of FgCnx-GFP expression strain under native promoter |
| FgCnx-GFP-NEST-F  FgCnx-GFP-NEST-R | AAGTGTGCCGATGCCTCTGG  CGATGGATCCCACGGTTCT | PCR primers to amplify the up-GFP-G418-down fusion fragment for the construction of FgCnx-GFP expression strain under native promoter |
| FgCnx-GFP-ID-F  FgCnx-GFP-ID-R | CTGTCCTCTTCAACCTTGGC  GTTGAGACAAGTCAGCCTTA | PCR primers for identification of correct np-FgCnx-GFP transformants |
| FgCnx-gpda-up-F  FgCnx-gpda-up-R | CGACAGGACGGAAGGATTTAAT  CAAAATAGGCATTGATGTGTTGACCTCCCTTAGGTACTTCTGGAGACCT | PCR primers to amplify upstream fragment flanking the native promoter of *FgCNX* for the construction of FgCnx-GFP expression strain under gpda promoter |
| FgCnx-gpda-down-F  FgCnx-gpda-down-R | CTACCCCGCTTGAGCAGACATCACCATGAAGTTAAACGCTGTCGCG  GCTTCCTCATCCCAGTCCTC | PCR primers to amplify downstream fragment flanking the native promoter of *FgCNX* for the construction of FgCnx-GFP expression strain under gpda promoter |
| FgCnx-gpda-nest-F  FgCnx-gpda-nest-R | CTAGAGCGGTAATGATTGGAGT  GCTGCCAGTCTTGACTTCCTC | PCR primers to amplify the up-HPH-gpda-down fusion fragment for the construction of FgCnx-GFP expression strain under gpda promoter |
| FgCnx-gpda-id-F  FgCnx-gpda-id-R | GTCCGGCTTAGTAGGTTGAATTG  GAAGCCTCCTTAACGTCCTCA | PCR primers for identification of correct gpda-FgCnx-GFP transformants |
| FfHmr1-GFP-up-F  FfHmr1-GFP-up-R | GCTGGTACCAACCTGTACATTCG  TGAACAGCTCCTCGCCCTTGCTCACTCGCTTTGACCTCTGAATAGC | PCR primers to amplify upstream fragment flanking the stop codon of *FfHMR1* for the construction of FfHmr1-GFP expression strain under native promoter |
| FfHmr1-GFP-down-F  FfHmr1-GFP-down-R | CTATCGCCTTCTTGACGAGTTCTTCTGAACGAATAACTTTGCATTTGAAGGAG  CTAACATCTCCAGTGCGGCTC | PCR primers to amplify downstream fragment flanking the stop codon of *FfHMR1* for the construction of FfHmr1-GFP expression strain under native promoter |
| FfHmr1- GFP-nest-F  FfHmr1- GFP-nest-R | CTAAGCGTCATGGCCAACGAT  ACTTCCACCCACCTTCCCACA | PCR primers to amplify the up-GFP-G418-down fusion fragment for the construction of FfHmr1-GFP expression strain under native promoter |
| FfHmr1-GFP-id-F  FfHmr1-GFP-id-R | GTCATCTCGTCCGAGCTCAC  AGTGGCAAGGGTGAAGACATAT | PCR primers for identification of correct np-FfHmr1-GFP transformants |
| FfHmr1-gpda-up-F  FfHmr1-gpda-up-R | AGCCTCAATGGTGATGGTGG  CAAAATAGGCATTGATGTGTTGACCTCCCCGCGTGGGTTACAGGGAAG | PCR primers to amplify upstream fragment flanking the native promoter of *FfHMR1* for the construction of FfHmr1-GFP expression strain under gpda promoter |
| FfHmr1-gpda-down-F  FfHmr1-gpda-down-R | CTACCCCGCTTGAGCAGACATCACCATGGCTGCGATTCTCCTGCC  CAGCTTCGTGGTGACGACGA | PCR primers to amplify downstream fragment flanking the native promoter of *FfHMR1* for the construction of FfHmr1-GFP expression strain under gpda promoter |
| FfHmr1-gpda-nest-F  FfHmr1-gpda-nest-R | GGGCGAAACGCCAAGACCAT  GTACAGATGCCGAGCCAGAAC | PCR primers to amplify the up-HPH-gpda-down fusion fragment for the construction of FfHmr1-GFP expression strain under gpda promoter |
| FfHmr1-gpda-id-F  FfHmr1-gpda-id-R | CTCGCATGATGATCGCCCTG  GGCCCATGACGGCGTAGTTT | PCR primers for identification of correct gpda-FfHmr1-GFP transformants |
| FoHmr1-GFP-up-F  FoHmr1-GFP-up-R | CGATTCAAGACCACCACCGG  TGAACAGCTCCTCGCCCTTGCTCACTCGCTTTGACCTCTGAATAGCAG | PCR primers to amplify upstream fragment flanking the stop codon of *FoHMR1* for the construction of FoHmr1-GFP expression strain under native promoter |
| FoHmr1-GFP-down-F  FoHmr1-GFP-down-R | CTATCGCCTTCTTGACGAGTTCTTCTGAACGAATAACTTTGCATTTGAAGGAG  ACCACCTAACAACCCTCCCCT | PCR primers to amplify downstream fragment flanking the stop codon of *FoHMR1* for the construction of FoHmr1-GFP expression strain under native promoter |
| FoHmr1-GFP-nest-F  FoHmr1-GFP-nest-R | CGTCATGGCCAACGATGGAG  CCCATCCTCCCACATATCCTT | PCR primers to amplify the up-GFP-G418-down fusion fragment for the construction of FoHmr1-GFP expression strain under native promoter |
| FoHmr1-GFP-id-F  FoHmr1-GFP-id-R | CGCAGGCGAGCTTTCTCTCT  CCATCTTTCGACGGCTGTATAT | PCR primers for identification of correct np-FoHmr1-GFP transformants |
| FoHmr1-gpda-up-F  FoHmr1-gpda-up-R | GAACCTCGGCTAAAGACATCG  CAAAATAGGCATTGATGTGTTGACCTCCGCACGGGCTTGAGGTGTCT | PCR primers to amplify upstream fragment flanking the native promoter of *FoHMR1* for the construction of FoHmr1-GFP expression strain under gpda promoter |
| FoHmr1-gpda-down-F  FoHmr1-gpda-down-R | CTACCCCGCTTGAGCAGACATCACCATGGCTTCGATTCTTCTGCCG  GGGAGACCCTCAGACAGAAG | PCR primers to amplify downstream fragment flanking the native promoter of *FoHMR1* for the construction of FoHmr1-GFP expression strain under gpda promoter |
| FoHmr1-gpda-nest-F  FoHmr1-gpda-nest-R | GCCGAAATCGCAGCTTTGCC  ACAGAGTGCAGACACCGAGC | PCR primers to amplify the up-HPH-gpda-down fusion fragment for the construction of FoHmr1-GFP expression strain under gpda promoter |
| FoHmr1-gpda-id-F  FoHmr1-gpda-id-R | CGTGGCATGGTTACAGTCGT  AAGTCGCTTGGAGGCCCATG | PCR primers for identification of correct gpda-FoHmr1-GFP transformants |
| FgGpmk1-UP-F  FgGpmk1-UP-R | GCATGCTGCTCTCATTCTTGTC  CAAAATAGGCATTGATGTGTTGACCTCCGGTCGCTGTGGTGGATGTGAT | PCR primers to amplify *FgGPMK1* upstream fragment for the construction of *FgGPMK1* deletion mutants |
| FgGpmk1-DOWN-F  FgGpmk1-DOWN-R | CTCGTCCGAGGGCAAAGGAATAGAGTAGCTCTGAAGCACCCATACCTTG  GCATGGAATGGCTCTGACACA | PCR primers to amplify *FgGPMK1* downstream fragment for the construction of *FgGPMK1* deletion mutants |
| FgGpmk1-NEST-F  FgGpmk1-NEST-R | CCCATTCCGCCCTGTTTCAT  GAGATGTAGGGAGCTAGTGGA | PCR primers to amplify *FgGPMK1* deletion fragment for the construction of *FgGPMK1* deletion mutants |
| FgGpmk1-ID-F  FgGpmk1-ID-R | AATCCTTCCGCGCCTTCTGT  GACTGGTGATTCTGTATGCGC | PCR primers for identification of *FgGPMK1* deletion transformants |
| FgMgv1-UP-F  FgMgv1-UP-R | TCGCTAATTCTGACCTGTTGCG  CAAAATAGGCATTGATGTGTTGACCTCCGGTGTATTTGTGTGGTGGTGTA | PCR primers to amplify *FgMGV1* upstream fragment for the construction of *FgMGV1* deletion mutants |
| FgMgv1-DOWN-F  FgMgv1-DOWN-R | CTCGTCCGAGGGCAAAGGAATAGAGTAGCTACGCAAAGTACAAGGGTTG  GGCGAGCCTACTGACACTGAA | PCR primers to amplify *FgMGV1* downstream fragment for the construction of *FgMGV1* deletion mutants |
| FgMgv1-NEST-F  FgMgv1-NEST-R | TGATGCCGAAGCCAGACAGG  CCGATGAGGAAGAAGAGGACA | PCR primers to amplify *FgMGV1* deletion fragment for the construction of *FgMGV1* deletion mutants |
| FgMgv1-ID-F  FgMgv1-ID-R | CACTGTCACTGCATTGCTCC  CCTCAGCCTGCTGAAGCCAA | PCR primers for identification of *FgMGV1* deletion transformants |
| FgHog1-UP-F  FgHog1-UP-R | GGGTTCAGGCTTATGGTGCCC  CAAAATAGGCATTGATGTGTTGACCTCCGGTGAATATGTGGTTGTTGAGCGAG | PCR primers to amplify *FgHOG1* upstream fragment for the construction of *FgHOG1* deletion mutants |
| FgHog1-Down-F  FgHog1-Down-R | CTCGTCCGAGGGCAAAGGAATAGAGTAGAAGGGAGAAGTGGTTAATAAAGGGG  GAAAGCTGCTCTTGAAACGTTCG | PCR primers to amplify *FgHOG1* downstream fragment for the construction of *FgHOG1* deletion mutants |
| FgHog1-NEST-F  FgHog1-NEST-R | GACAAGACCCAGTCTTCTGTTGTC  CGAATAATGGATATACAGAGCGG | PCR primers to amplify *FgHOG1* deletion fragment for the construction of *FgHOG1* deletion mutants |
| FgHog1-ID-F  FgHog1-ID-R | CCTGTTCCTCGTTCTGCAGC  GGGCGTATGCGTTGAATTTCG | PCR primers for identification of *FgHOG1* deletion transformants |
| FgHac1-intron-F  FgHac1-intron-R | TTGAAGAGATCGCCGAACAG  GAAAGCAGCATCGCCTGGAA | PCR primers used for RT-PCR to amplify *FgHAC1* mRNA fragment that contains the 20-bp unconventional intron |
